# Stress transmission along mid-crustal faults highlighted by the 2021 Mw 6.5 San Juan (Argentina) earthquake

**DOI:** 10.1038/s41598-022-22752-6

**Published:** 2022-10-26

**Authors:** Jean-Baptiste Ammirati, Chelsea Mackaman-Lofland, Martin Zeckra, Kevin Gobron

**Affiliations:** 1 Departamento de Geología, Facultad de Ciencias Físicas y Matemática, Universidad de Chile, Plaza Ercilla 803, 8320000 Santiago, Chile; 2 Department of Geosciences, University of Connecticut, Beach Hall, 354 Mansfield Rd 207, Storrs, CT 06269 USA; 3 Department of Earth and Environmental Sciences, Denison University, 100 West College St, Granville, OH 43023 USA; 4 Royal Observatory of Belgium, Avenue Circulaire 3, 1180 Uccle, Belgium; 5 Institut de Physique du Globe de Paris, CNRS, IGN, Université de Paris Cité, 75005 Paris, France; 6 Present Address: Institute of Earth Sciences, University of Lausanne, 1015 Lausanne, Switzerland

**Keywords:** Seismology, Tectonics

## Abstract

Understanding the mechanisms of crustal deformation along convergent margins is critical to identifying seismogenic structures and assessing earthquake hazards for nearby urban centers. In the southern central Andes (28–33$^{\circ }$S), differences in the style of middle to upper-crustal deformation and associated seismicity are highlighted by the January 19th, 2021 (Mw 6.5) San Juan earthquake. We integrate waveforms recorded at regional and teleseismic distances with co-seismic displacements calculated from local Global Navigation Satellite System time series, to re-estimate the source parameters of the 2021 San Juan earthquake, confirming a mid-crustal nucleation depth (21 ± 2 km) and right-lateral transpressional mechanism. Considered alongside decades of seismic observations and geological data, this event provides evidence for retroarc deformation partitioning among inherited basement faults and upper-crustal structures in response to oblique convergence of the Nazca and South American plates. As they may transfer shortening to active upper-crustal faults associated with historically devastating shallower earthquakes, a better understanding of seismogenic basement faults such as the mid-crustal structure activated during the 2021 San Juan earthquake earthquake could help future re-assessment of the seismic risk in western Argentina.

## Introduction

Variations in the morphology, seismicity, and deformation style of Cordilleran orogens (e.g. Andes, North American Cordillera) are commonly attributed to changes in ocean-continent plate convergence. Oblique subduction induces the partitioning of deformation into trench-normal and trench-parallel components that may be resolved by oceanic plate consumption and/or localized strike-slip or transpressional faulting in the overriding plate^1^. Changes in subduction zone geometry, such as flattening of the oceanic slab, are moreover associated with enhanced plate coupling, elevated seismicity, and basement-involved deformation expressed up to 700–1500 km inboard of the trench^2–4^. The resulting basement uplifts may be characterized by variable structural orientations and complex relationships with the retroarc fold-thrust belt, precursor basement features, plate convergence dynamics, and regional stress field^5–7^. Understanding the mechanisms and connections governing stress transmission, deformation, and seismicity remains particularly challenging in retroarc systems involving both fold-thrust belts (characterized by ramp-flat fault styles above a regional décollement) and basement-involved intraforeland structures (which penetrate middle to lower crustal levels^8–12^, with implications for the location and distribution of major seismogenic faults.

**Figure 1:**
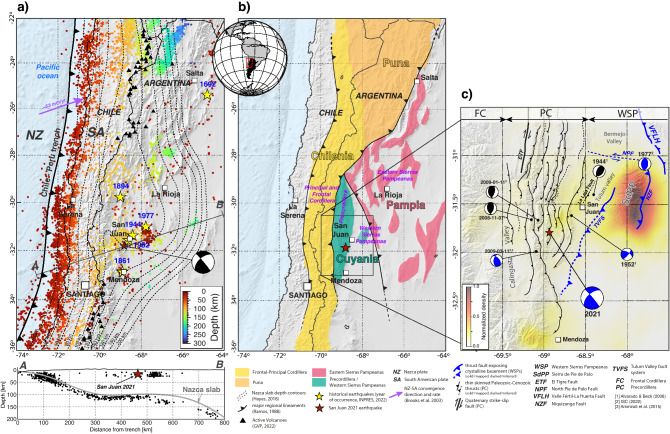
General seismotectonic context of the western margin of South America. (**a**) Map showing the distribution of seismicity from the 1964–2018 EHB-Catalog^13^. Dashed lines illustrate contours to top of the Nazca oceanic slab after the SLAB 2.0 model^16^. Black triangles show Holocene active volcanoes. Yellow stars indicate epicenters of major historical retroarc earthquakes^14^. The red star shows the location of the 2021 San Juan earthquake and corresponding focal mechanism^15^. The purple arrow shows the Nazca-South America convergence velocity rate (fixed South America reference frame). The lower cross section shows seismicity projected onto section line A–B; gray line in the cross section corresponds to the top of the Nazca oceanic plate following^16^. (**b**) Map highlighting the main structural features and retroarc domains of the southern central Andes^17^. (**c**) Regional map of the study area showing main retroarc structures and associated crustal seismicity above the Chilean-Argentinian flat-slab subduction segment^13,18^. Focal mechanism solutions are shown for regionally significant earthquakes at middle-crustal (blue) or upper-crustal (black) focal depths. This map was created using the GMT package (V.6.0.0, https://www.generic-mapping-tools.org/). Topographic information corresponds to the Shuttle Radar Topographic Mission 90m database (https://srtm.csi.cgiar.org/srtmdata/) and the ETOPO1 Global Relief Model (https://www.ngdc.noaa.gov/mgg/global/).

Between 28$^{\circ }$S and 33$^{\circ }$S, the Andes of western Argentina define superb examples of overlapping fold-thrust belt and basement-involved retroarc deformation above a zone of flat-slab subduction, and are considered a modern analogue to the archetypal Sevier and Laramide belts of the North American Cordillera^3,12,19^. Above the Chilean-Argentinian flat-slab subduction segment (28–33$^{\circ }$S), the Andes are characterized by a narrow, N-trending retroarc fold-thrust belt that parallels the subduction trench (Precordillera), NW- to NE-trending intraforeland basement uplifts that expand up to $\sim$ 700 km toward the craton (Sierras Pampeanas), and high levels of crustal seismicity in both downgoing and overriding plates^4^ (Fig. 1). These retroarc features are the product of multiple orogenic cycles, including: (1) the Ordovician-Early Carboniferous Famatinian orogeny, characterized by accretion of the Pampia, Cuyania, and Chilenia terranes; (2) Carboniferous-early Triassic regional extension; and (3) the Neogene-present day Andean orogeny, which generated the modern Precordillera and Sierras Pampeanas^17^ (Fig. 1). Shortening in the Western (WPC) and Central Precordillera (CPC) occurred during middle to late Miocene flattening of the subducted slab and formed a N-striking, E-directed thrust belt involving Paleozoic-Cenozoic sedimentary cover^6,20^. The W-directed Eastern Precordillera (EPC) also exposes Paleozoic-Cenozoic sedimentary rocks; construction of the EPC is documented by the late Quaternary and structures show evidence of active deformation^21^. The Sierras Pampeanas exhume crystalline basement, notably within the NE-trending Sierra de Pie de Palo (SdPP) and along the NW-striking, E-dipping Valle Fértil-La Huerta fault (VFLH) that defines the reactivated suture zone between Cuyania and Pampia terranes^6,22^.

Numerous retroarc earthquakes have been documented^14^ above the Chilean-Argentinian flat-slab segment, including the devastating 1861 Mendoza ($Mw=7.2$) and 1944 San Juan ($Mw=7.0$) earthquakes, followed by the 1952 ($Mw=6.8$) and 1977 ($Mw=7.4$) events near the city of San Juan (Fig. 1a, c). While most of these devastating earthquakes occurred beneath the NE-striking EPC and SdPP^23^ (Fig. 1), considerable uncertainty persists in both the continuation and interactions of EPC and SdPP structures at depth^11,24,25^. Seismic monitoring over the past several decades indicates that most retroarc earthquakes are located between 5 and 30 km depth, and are generally characterized by reverse and/or strike-slip focal mechanisms^26–29^. Considering a South America fixed reference frame, the convergence of the Nazca and South American plates in the region of flat-slab subduction (28–33$^{\circ }$S) occurs at 63 mm/yr in the $\sim$N70$^{\circ }$ direction^30^. Earthquake focal mechanisms and wellbore breakouts suggest a broadly W-E orientation of the regional compressive stress^10,31,32^.

On January 19th, 2021 (2:46 UTC), San Juan was shaken by a Magnitude 6.5 earthquake (Fig. 1), the largest crustal event to affect the Argentine retroarc since 1977. Epicenter solutions estimated by the Argentine Institute for Seismic Prevention (INPRES) and other agencies indicate the 2021 San Juan earthquake (hereafter SJ2021) occurred $\sim$50 km southwest of San Juan. Published focal mechanisms consistently show a strike-slip reverse solution with nodal planes oblique to structures in the overlying, N-trending Precordillera thrust belt (Fig. 1, Table S1 and references therein). The estimated focal depth, however, varies considerably: local solutions from INPRES and the Chilean Seismological Center (CSN) are characterized by shallow depths of 8–10 km, whereas teleseismic observations from international agencies point to a much deeper focal depth of $\sim$17–25 km^15^. Using local data available from INPRES, Girino et al.^33^ re-estimated the focal depth of the SJ2021 to an even shallower 5 ± 7 km, though this estimate is subject to a significant uncertainty. These authors also used first-motion polarities to constrain the SJ2021 focal mechanism, yielding results consistent with the focal mechanism solution obtained by international agencies (Fig. 1, Table S1).

Given their respective uncertainty level, the focal depth discrepancy between Girino et al.^33^ local solution (5 ± 7 km) and teleseismic observations^15^ (21 ± 4 km) is significant, which is unusual for such a high magnitude event ($Mw=6.5$). Resolving this discrepancy is of particular importance because different focal depth estimations could lead to disparate tectonic interpretations of the structures accommodating active shortening. Therefore, accurate depth re-estimation of the SJ2021 is essential to understand how shortening and transpressional deformation are accommodated at middle to upper-crustal levels, and improve interpretations of the geometry, seismicity, and potential rupture lengths along emergent and subsurface faults.

As key information, including the seismic phases and waveforms recorded by nearby INPRES seismic stations remain unavailable, This study relies on seismic phase information from the ISC bulletin^13^ and waveform records obtained at regional and teleseismic ($\sim$100–10,000 km) distances. To better constrain the source parameters of the SJ2021, we invert its coseismic static displacement recorded by 4 nearby Global Navigation Satellite System (GNSS) stations, jointly with the regional and teleseismic waveforms. We then consider the event alongside crustal seismicity data and geological constraints to interrogate middle and upper-crustal deformation patterns at $\sim$31.5$^{\circ }$S. Then we explore the mechanisms and interactions controlling stress transmission and shortening along variably oriented structures associated with a zone of oblique plate convergence, ongoing flat-slab subduction, and high earthquake risk.

## The deep-crustal character of the San Juan 2021 earthquake

Despite the strong reported intensity and damage observed in several urbanized areas close to the epicenter^15^, no clear surface rupture was observed for the SJ2021^34^. These findings are consistent with the absence of significant surface deformation determined from Interferometric Synthetic Aperture Radar (InSAR) data (Fig. S2). Simple synthetic static displacement modeling of a magnitude 6.5 earthquake at 5 km depth (Fig. S3) predicts coseismic displacements greater than 40 cm in both horizontal and vertical directions. While the magnitude trigger for surface rupturing in such a compressive tectonic setting might be higher than the SJ2021 magnitude ($Mw=6.5$), these displacement values strongly suggest that a shallow SJ2021 should have generated surface displacements clearly discernable on the interferograms. Both field and InSAR observations are thus indicative of a deep-crustal character for the SJ2021.

**Figure 2:**
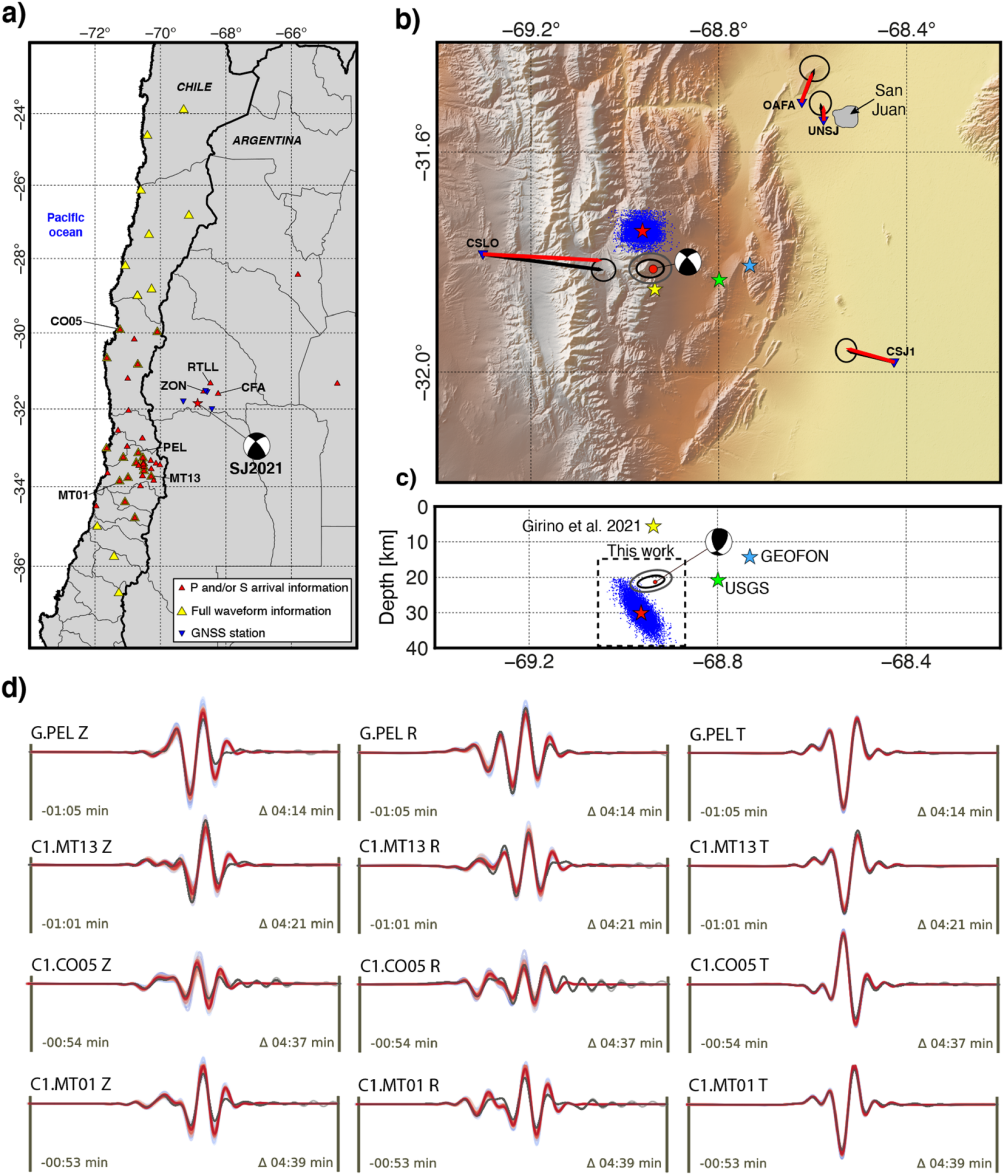
Refined source parameters of the 2021 San Juan earthquake. (**a**) Map showing the location of seismic and GNSS stations used in this study. Red triangles denote seismic stations used for earthquake relocation. Yellow triangles indicate stations for which the full waveform was used in inversion of the full moment tensor. Blue inverted triangles represent the GNSS stations used in this study. (**b**) Map showing epicentral locations for the 2021 San Juan earthquake (SJ2021) corresponding to the USGS^15^ solution (green star), GEOFON solution (blue star), Girino et al.^33^ (yellow star), our relocated epicenter (red star, with samples of the hypocenter probability density function shown as blue dots), and our final location and focal mechanism solution from the inversion of regional waveforms and GNSS coseismic displacement (beachball diagram and red dot). (**c**) Cross section view of the information presented in (**b**) projected onto section line A–B. Note that the SJ2021 focal mechanism solution has been rotated into the cross section plane. (**d**) Seismic waveforms corresponding to 4 example stations (see Fig. 2a for location) with observed surface waves (gray) after restitution, filtering, tapering and rotation into ZRT coordinate system, and corresponding synthetic waveforms ensemble (colored from blue to red with decreasing misfit). (**a**)–(**c**) were generated using the GMT package (V6.0.0, https://www.generic-mapping-tools.org/) The topographic information in (**c**) corresponds to the Shuttle Radar Topographic Mission 90m database (https://srtm.csi.cgiar.org/srtmdata/). (**d**) was generated using the Python Matplotlib libraries (V3.5.2, https://matplotlib.org/).

We attempt to resolve the persistent discrepancy in focal depth estimates from published solutions^15,33^ via a combination of techniques, including: (1) hypocenter re-estimation based on publicly available local and regional seismic phase information; (2) depth phase analysis using teleseismic records, a technique particularly well-adapted to constrain the focal depth of events for which near-field information is unavailable; and (3) full moment tensor inversion from the joint inversion of regional waveforms and static displacement from nearby GNSS stations. Combined, these techniques provide multiple avenues to resolve the focal depth and understand the reasons for the discrepancy in published estimates.

Accurate hypocenter location using local seismic data is usually achieved when three analytical conditions are met^35–37^: (1) A maximum angle between two adjacent seismic station (azimuthal gap) lower than 180$^{\circ }$; (2) A minimum of 8 seismic phases per event, of which at least one corresponds to an S-wave; and (3) at least one accurate S-wave phase recorded at short epicentral distance (typically 1.4 times the focal depth) from the epicenter. The configuration of the Argentine network^33^, combined with stations located in the Chilean central region^38^, provides dense coverage and a good azimuthal distribution of observations above the Chilean-Argentinian flat-slab (Fig. 2a). With an epicentral location $\sim$50 km southwest of the city of San Juan and $\sim$40 km southwest from the closest station (ZON, Fig. 2a), the SJ2021 meets the above conditions for a well-constrained hypocenter location using both local and distant observations. Given the excellent station coverage for the SJ2021, reasons for the significant focal depth discrepancy between the local^33^ (5 ± 7 km) and teleseismic solutions^15^ (21 ± 4 km) remain unclear.

We used events and phase information from the ISC bulletin^13^, including three phases picked from local seismic stations (per the analytical conditions described above; Fig. 2a) and a regionally calibrated velocity model^39^ to re-estimate the hypocenter location corresponding to the SJ2021. Results demonstrate that our solution is shifted to the northwest ($\sim$13 km) compared to the USGS location, and $\sim$4 km north the INPRES refined solution^33^ (Fig. 2b). The hypocenter depth is 30 ± 6 km, which is partly overlapping with the USGS estimate of 21 ± 4 km^15^.

**Figure 3:**
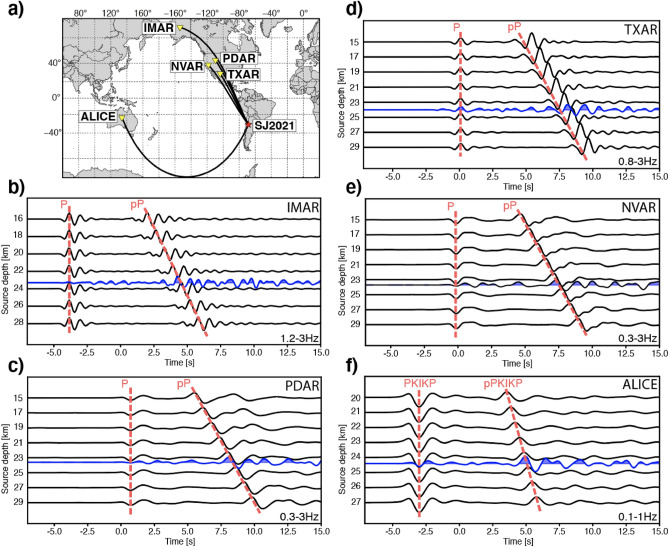
Plots comparing the synthetic (black) waveforms simulating different focal depths of the San Juan 2021 earthquake (SJ2021) and beamformed array recordings of the SJ2021 event (blue). The best fit between synthetic and observed waveforms is achieved for focal depths between 23 and 25 km. Red dashed lines denote direct P arrivals and corresponding depth phases. This figure was generated using the Python Matplotlib libraries (V3.5.2, https://matplotlib.org/). (**a**) was created using the GMT package (V.6.0.0, https://www.generic-mapping-tools.org/).

It is possible to further constrain the deep character of the SJ2021 by identifying near-source body wave reflections at the free surface (also called depth phases) at teleseismic distances and comparing the observed waveforms with a set of synthetic waveforms computed for several focal depths (Fig. 3). The Identification of depth phases stemming from intermediate magnitude (5$<Mw<$6) earthquakes may be challenging over large distances, so we enhanced depth phase signals using a beamforming approach wherein waveforms of seismic arrays are phase shifted and constructively stacked to amplify the signals. Our results demonstrate minor but persistent dissimilarities between the observed and synthetic waveforms, which may reflect inadequacies in the velocity models close to the source or at receiver locations, and/or local topographic effects of the free surface on the source side (Fig. 3). However, we emphasize that waveform polarities and the dominant frequencies are well reproduced by synthetic data generated from focal depths of 23–24 km, a depth range in good agreement with the USGS findings^15^ and our hypocenter solution (Figs. 2b, 3).

**Figure 4:**
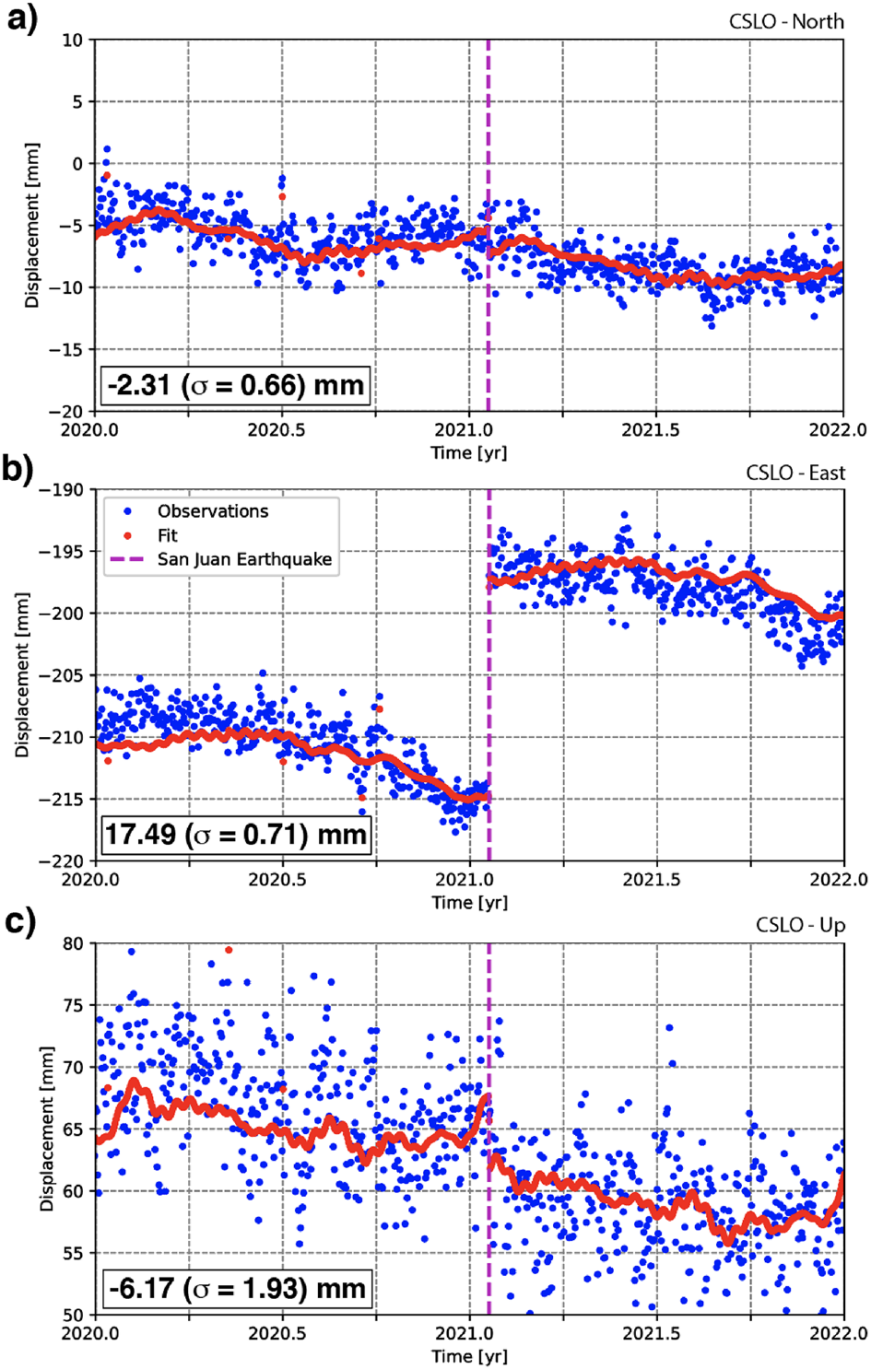
GNSS displacements recorded at station CSLO (see Fig. 2 for location) for years 2020 and 2021. The blue dots represent detrended measurements; red dots show the trajectory model (see text for description and interpretation). Consistent displacement along all three (north, east, and up) components is traceable to the date/time of the 2021 San Juan earthquake.

The surface coseismic displacement generated by the SJ2021 was recorded by four GNSS stations located within a radius of $\sim$50 km (Fig. 2, 4, Table S2). The displacement was relatively small, in general <10 mm except for the closest station (CLSO, located $\sim$35 km west of the SJ2021 epicenter), where an eastward displacement of $\sim$17 mm is clearly visible at the date of the earthquake (Fig. 2b, 4b, Figs. S6–S9). Note that coseismic displacements exceeding 3$\sigma$ (e.g., significant at the 99% confidence level) are observed for all four GNSS stations.

Finally, we jointly inverted these coseismic displacements with regional waveforms to compute the SJ2021 full moment tensor (Fig. 2b, c). The notable similarity between synthetic and observed waveforms (Fig. 2d) illustrates the robustness of the solution. Our inversion results indicate a centroid located at 31.812$^{\circ }$S, 68.935$^{\circ }$W and 21 ± 2 km depth. The right-lateral, transpressional focal mechanism is consistent with solutions published by the USGS^15^ and the INPRES^33^ (Table S1), and we suggest that the rupture occurred along the preferred fault plane characterized by a strike, dip, and rake of N43$^{\circ }$E, 66$^{\circ }$NW, and 163$^{\circ }$, respectively. The full report corresponding to the presented solution and associated uncertainty is publicly available^40^. It is interesting to note that our full moment tensor solution considerably diverge from a pure double-couple (DC) mechanism. This may indicate complex source mechanisms rupturing a multi-segmented and/or a non planar structure^41^. We also acknowledge that non-DC events can be associated with a poor coverage of seismic stations and reflect inadequacies in the velocity model used for the inversion^42^.

## Integrating retroarc seismicity with structural interpretations

**Figure 5:**
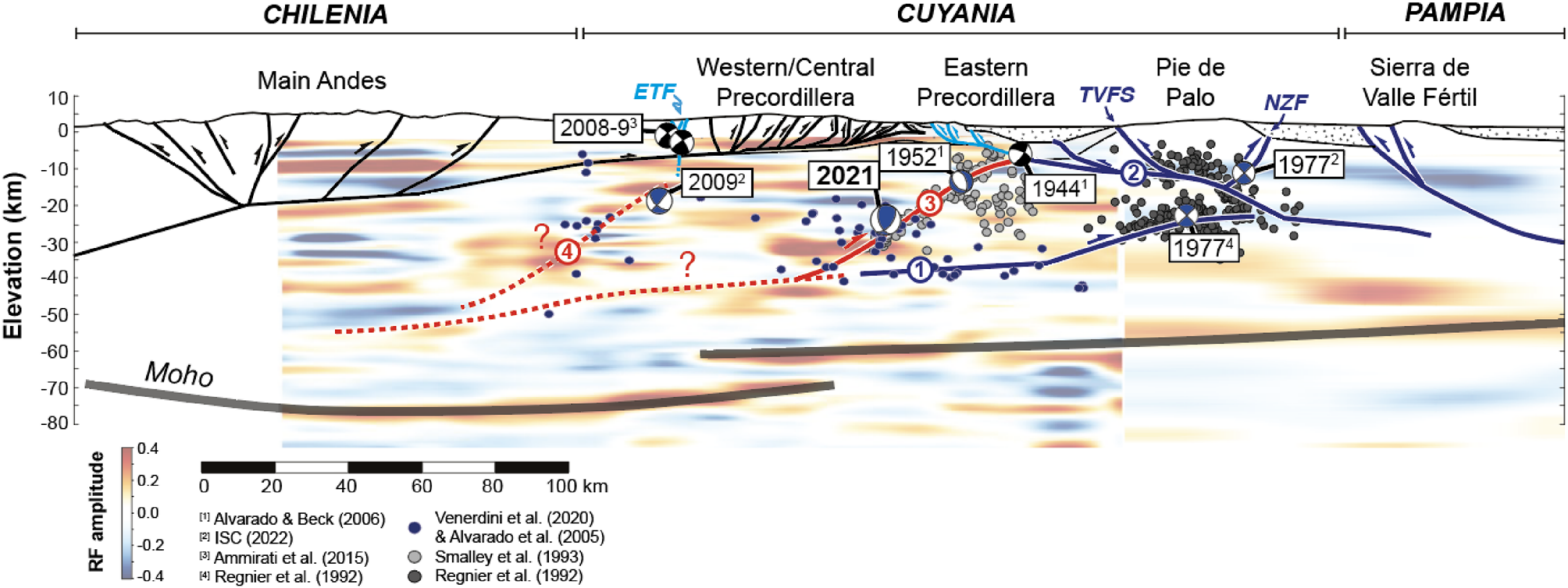
Crustal-scale cross section of the Andean retroarc at $\sim$31.5$^{\circ }$S highlighting regional seismicity (grey and blue circles), middle- to upper-crustal structures, and historically significant earthquake focal mechanisms. Emergent and subsurface fault geometries are modified from^6,9,11,18,20,25^. Receiver function data are from^39^. Fault-like structures inferred from geophysical data are marked with red and blue numbered circles and correspond to structural features described in the text. All earthquake focal mechanisms have been rotated into the cross section plane.

Our final, inverted SJ2021 hypocenter solution is plotted alongside historical earthquake hypocenters^13,23,39^, resulting from the^43^ Portable Array for Numerical Data Acquisition (PANDA, 1987–1988), SIEMBRA^44^ (Sierras Pampeanas Experiment using a Multicomponent Broadband Array, 2007–2009), and CHARGE^29^ (Chile-Argentina Geophysical Experiment, 2000–2002; Alvarado et al.^29^) temporary seismic experiments, as well as receiver function geophysical data^45^, to illustrate patterns of retroarc seismicity along a cross section at $\sim$31.5$^{\circ }$S (Fig. 5). Seismicity beneath the Western Precordillera and Central Precordillera is concentrated between 20 and 40 km depths. With the exception of several events in the Western Precordillera, no activity is observed within the thin-skinned thrust belt (i.e., within the closely-spaced faults above the shallow décollement at $\sim$10 km depth^32^). Relatively intense seismicity is documented beneath the Eastern Precordillera and Sierra de pie de Palo (SdPP) between 5 and 35 km depths, including the 1944, 1952, and 1977 historical earthquakes and the SJ2021^9,10,13,23,26–28^ (Fig. 1c).

Previous interpretations of fault-like structures from these datasets include (Fig. 5): (1) A décollement at $\sim$20–40 km depth that may connect basement structures beneath the Precordillera and SdPP^32,45^; (2) An upper subhorizontal to E-dipping fault in the SdPP at $\sim$10–15 km depth. This fault has been interpreted as the passive roof duplex of an E-directed middle crustal wedge^6^, or the back thrust of a listric, E-directed basement thrust fault inferred beneath the central and eastern SdPP^25,26^. In either case, the upper subhorizontal to E-dipping, W-directed SdPP fault may be kinematically linked to emergent faults in the Eastern Precordillera^11,18^; (3) A N- to NE-striking, NW-dipping basement fault beneath the Eastern and Central Precordillera^27^; (4) Other generally W-dipping basement fault planes inferred from diffuse microseismicity beneath the Central and Western Precordillera^32^. We note that the basement faults inferred from deep Precordillera seismicity overlap W-dipping, high amplitude arrivals in the receiver function data, and the regional décollement may coincide with a gently west-dipping, low amplitude arrival in the middle to lower crust^45^ (Fig. 4). As décollements preferentially localize at weak crustal levels and ramps commonly occur in more competent intervals, these observations suggest that the receiver function data track changes in rock properties that influence subsurface structural geometries.

Relating earthquakes to specific faults remains challenging due to persistent uncertainties in event locations and along-strike variations in the orientation of both emergent and subsurface structures, making planar faults difficult to interpret from small-magnitude seismicity data^26,28,29,46^. Even so, our study builds upon careful integration of historical earthquakes with the structural framework of the Precordillera and Sierras Pampeanas. For example, Alvarado and Beck^23^ linked the devastating 1944 San Juan earthquake to upward rupture along the La Laja fault (Eastern Precordillera) based on the excellent match between their preferred fault plane solution (strike N45$^{\circ }$E, dip 35$^{\circ }$SE, rake 110$^{\circ }$) and fault parameters measured on the 1944 La Laja scarp (strike N45$^{\circ }$E, dip 25–45$^{\circ }$SE, rake 90$^{\circ }$)^47^, as well as the correspondence between the La Laja surface rupture and surface displacement predicted from their best-fit shallow ($\sim$11 km depth) hypocenter location (Figs. 1c, 5). Persistent uncertainties in the epicentral location of the 1952 earthquake have prevented association with a discrete structure, but the reverse strike-slip focal mechanism (strike N40$^{\circ }$E, dip 75$^{\circ }$SE, rake 30$^{\circ }$) and best-fit focal depth range (10–13 km) are consistent with rupture along emergent NE-striking faults in the Eastern Precordillera^23^. Left-lateral neotectonic displacements identified along these Eastern Precordillera faults are further compatible with the oblique focal mechanism solution for the 1952 event^23^. The geometry of faults activated during the 1977 San Juan earthquake remains debated. However, the focal mechanism and depth solutions for the foreshock and main shock (pure thrust focal mechanisms with N-striking nodal planes and depths of $\sim$17 km and 25–30 km, respectively), and pattern of aftershock events are consistent with the activation of two fault planes of potentially opposing dips beneath the SdPP^25,26,28,48,49^.

**Figure 6:**
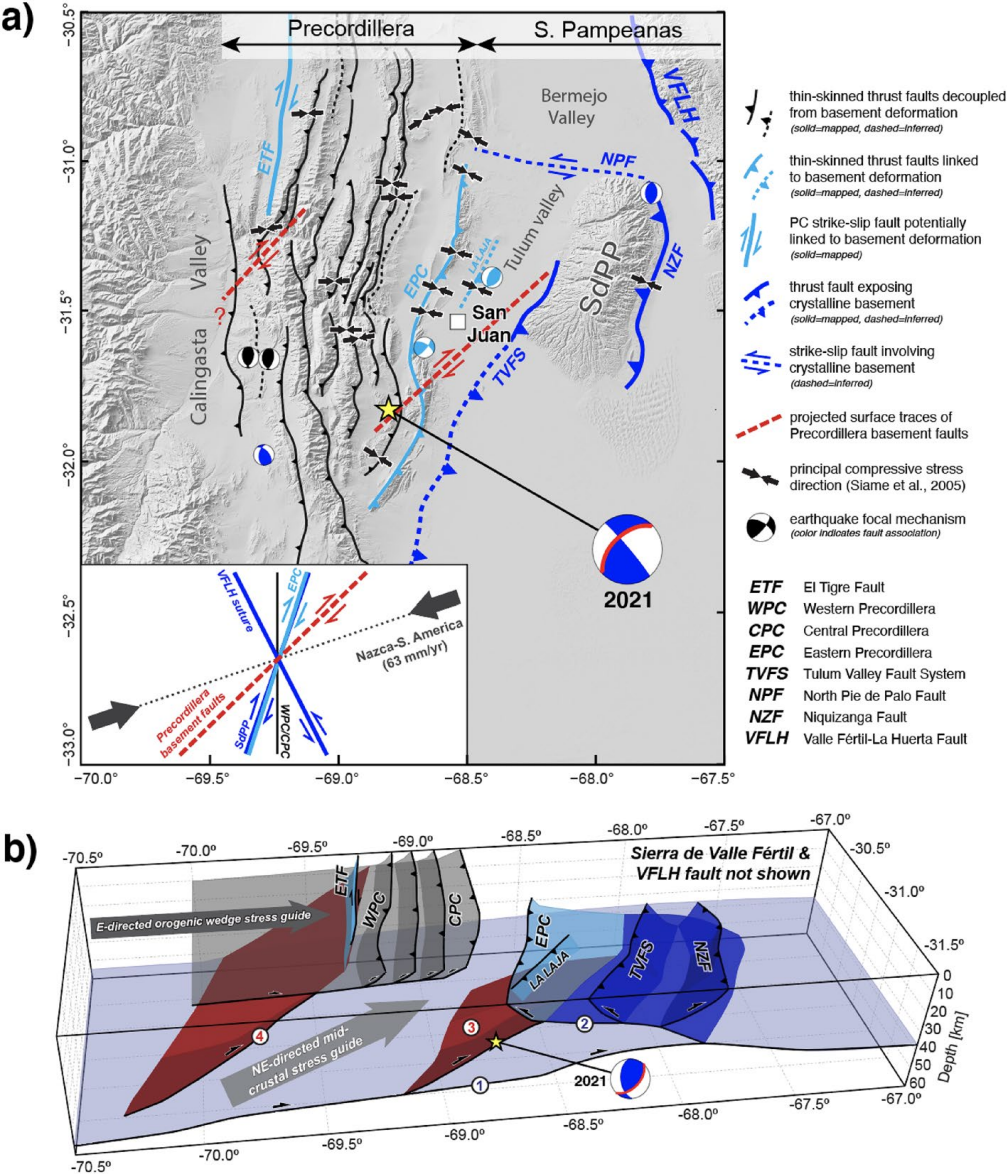
(**a**) Regional map illustrating middle- to upper-crustal interactions and inferred kinematic affiliations among retroarc structures. Red dashed lines show projected surface intercepts of Precordillera basement faults inferred from microseismicity data^27,32^. Black arrows show direction of principal compressive stress estimated from fault slip data^10^. Bottom inset depicts predicted Neogene-Quaternary partitioning of the  N70$^{\circ }$E plate convergence vector along variably oriented, middle- to upper-crustal retroarc features (modified from^10,27,30,63^. (**b**) Schematic block diagram showing proposed relationships among NE-striking, probably inherited basement faults and emergent structures in the Precordillera and western Sierras Pampeanas, in a South America fixed reference frame. Fault-like structures inferred from geophysical data are marked with red and blue numbered circles and correspond to structural features described in the text. The Note that the SJ2021 focal mechanism has been rotated into the southern cross section plane. This figure was created with the GMT package (V.6.0.0, https://www.generic-mapping-tools.org/. Topographic information in **a**) comes from the Shuttle Radar Topographic Mission 90 m database (https://srtm.csi.cgiar.org/srtmdata/).

In this context, we emphasize that our depth solution for the SJ2021 coincides with the NE-striking, NW-dipping basement fault defined by the zone of high seismic activity beneath the CPC and EPC (structural feature N$^{\circ }$3 in Fig. 5), and the preferred rupture plane (strike N43$^{\circ }$E, dip 66$^{\circ }$NW, rake 163$^{\circ }$) nearly matches fault parameters estimated from a least squares fit of the Precordillera basement hypocenter locations (N45$^{\circ }$E strike and 35$^{\circ }$NW dip, Figs. 5; 6a; Smalley et al.^27^). Moreover, we reiterate that the lack of an observed surface rupture strongly supports our deep 21 ± 2 km depth solution. We speculate that the steeper dip of the SJ2021 focal mechanism solution may result from three-dimensional changes in fault orientation that were not captured by the microseismicity data, and propose that the SJ2021 activated the NW-dipping Precordillera basement fault previously identified by Smalley et al.^27^. This non-emergent structure may be kinematically connected to the upper subhorizontal to E-dipping fault inferred beneath the SdPP (structural feature N$^{\circ }$2; Fig. 5) or transfer shortening directly to W and NW-directed active thrusts expressed in the Eastern Precordillera (including the La Laja fault, Fig. 1c)^11,18,23,24,50^. Altogether, the SJ2021 and other patterns of retroarc seismicity highlight the role of both middle and upper-crustal structures in accommodating active deformation and generating large magnitude earthquakes above the Chilean-Argentinian flat-slab segment.

## Stress transmission along basement and upper-crustal structures

Differences in structural geology and orientation in the analogous Sevier fold-thrust belt and Laramide basement provinces in western North America have been variably attributed to temporal rotations in the direction of principal compressive stress^51^, partitioning of oblique, unidirectional compressive stress along strike-slip faults or low-angle lateral ramps^7,52^, the reactivation of inherited basement weaknesses or conjugate faults^53,54^, and/or differing zones and modes of stress transmission in the orogenic wedge and middle crust^5,8,12^. The SJ2021, historical seismic events, and published structural data at $\sim$31.5$^{\circ }$S provide opportunities to interrogate the mechanisms and stress conditions controlling retroarc deformation in an active flat-slab subduction setting.

The Nazca-South America plate convergence vector has remained relatively constant during Neogene-Quaternary construction of the Precordillera and Sierras Pampeanas^30,55,56^, precluding changes in the direction of principal compressive stress as a function of plate dynamics. Fault slip data from N-striking Werstern and Central Precordillera thrust sheets further indicate reverse dip-slip motion and a W-E orientation of principal compressive stress since Neogene times^10^. Orientation of the thin-skinned Western and Central Precordillera thrust belts more closely align with the N-striking subduction margin than the $\sim$N70$^{\circ }$E direction of plate convergence^10,30^, a minor ($\sim$20$^{\circ }$) obliquity that may reflect the influence of topographic gravitational load on stress transmission within the orogenic wedge, and/or partitioning of the plate convergence vector into strike-slip and dip-slip components^8,12,57,58^. In support of the latter, we note that strike-slip displacement has been documented in Quaternary deposits along the N-striking, right-lateral El Tigre Fault (ETF, Fig. 1c, 5) expressed west of the Western Precordillera^10,59,60^.

Precursor basement faults influenced the construction and orientation of key ranges in the Sierras Pampeanas, notably including uplift of the Sierra de Valle fértil along the reactivated,  N30$^{\circ }$W-striking Cuyania-Pampia suture zone (VFLH Fault; Figs. 1, 5, 6)^6,61^. Partitioning of the modern plate convergence vector would be expected to produce compression and possibly left-lateral transpression along a structure of this orientation (Fig. 6a). These conditions are supported by the predominance of reverse dip-slip kinematic indicators along the VFLH Fault and minor left-lateral strike-slip faulting in the northern Sierra de Valle Fértil^10^. Other basement faults in the study area, including the Niquizanga Fault (NZF) and Tulum Valley Fault System (TVFS), at the margins of the SdPP, the NW-dipping basement fault associated with the SJ2021 (structural feature N$^{\circ }$3, Figs. 5, 6), and the other, generally W-dipping fault inferred beneath the Central and Werstern Precordillera (structural feature N$^{\circ }$4; Figs. 1, 5, and 6)-have not been directly linked to structural inheritance. Yet, we emphasize that the NZF, TVFS, and SJ2021 fault systematically strike $\sim$N20$^{\circ }$E to $\sim$N45$^{\circ }$E, within the range of orientations expected for structures conjugate to the Paleozoic VFLH suture^5,54^. These basement faults notably parallel the $\sim$N20$^{\circ }$E-striking Eastern Precordillera, which exposes Paleozoic-Cenozoic sedimentary rocks (Fig. 6a).

Compressive stress in the direction of plate convergence would be expected to generate right-lateral transpression along the aforementioned NE-striking Eastern Precordillera and SdPP features. While this prediction is consistent with the right-lateral focal mechanism calculated for the deep SJ2021, it is at odds with the pure thrust solutions obtained for the shallow 1944 and double-rupture 1977 earthquakes, and with fault slip data indicative of dip-slip thrusting and N100$^{\circ }$E- to N120$^{\circ }$E-directed Neogene-Quaternary compressive stress in the Easter Precordillera and SdPP^9,10,23^. Significantly, these discrepancies in orientation and stress conditions among Precordillera and Sierras Pampeanas structures are consistent with discrete zones and mechanisms of middle- and upper-crustal stress transmission originally hypothesized for the Sevier-Laramide flat slab segment^8^, and allow us to propose a model for retroarc deformation characterized by the following (Fig. 6b): (1) Basement-involved deformation is driven by a middle-crustal stress guide linked to enhanced plate coupling and stress loading of the lower continental lithosphere^5,8^. The resulting compressive stress is parallel to NE-directed plate convergence and accommodated via left- or right-lateral transpression along systematically NW- and NE-striking, probably reactivated basement faults^6^; (2) Transpression may be largely accommodated within the middle crust, resulting in clockwise rotation of the stress orientation from middle to upper structural levels. Clockwise rotation of the retroarc stress field is supported by GNSS data and further accommodated by left-lateral slip along the WNW-ESE-striking, basement-involved North Pie de Palo Fault^30^ (NPF, Figs. 1, 6a); (3) Deformation in the Western and Central Precordillera fold-thrust belt was controlled by an upper-crustal stress guide modulated by topographic body forces within the orogenic wedge^8,12^. The resulting compressive stress was perpendicular to the subduction margin and generated N-striking, thin-skinned thrusts. We emphasize that while the EPC exhumes the same Paleozoic-Cenozoic sedimentary cover rocks as the Western and Central Precordillera, its $\sim$N20$^{\circ }$E orientation strongly supports kinematic linkages with underlying, NE-striking basement features such as the SJ2021 fault (structural feature N$^{\circ }$3) or structures beneath the SdPP.

Finally, while active thrusting has ceased in the Western and Central Precordillera, persistent middle-crustal seismicity beneath these domains may highlight important linkages between other basement structures such the generally W-dipping basement fault inferred from diffuse microseismicity data (structural feature N$^{\circ }$4; Figs. 5, 6) and seismically active upper-crustal faults^32^. Thus, in addition to the connections posed between NE-striking basement faults, emergent structures in the Eastern Precordillera and SdPP, and historical large-magnitude earthquakes (1944, 1952, 1977, and 2021), we do not exclude further linkages with the western basement fault that may transfer right-lateral displacement to the Quaternary ETF^27,32,62^ (Figs. 5, 6). Coseismic activation of all of these emergent structures (ETF, Eastern Precordillera, and SdPP faults) may be anticipated during future occurrences of high-magnitude, middle-crustal earthquakes such as the SJ2021.

## Conclusions

We integrate seismic phase information from the ISC bulletin (ISC^13^), waveform records from regional and teleseismic distances, and local GNSS observations to re-estimate source parameters for the January 19th, 2021 ($Mw=6.5$) San Juan earthquake. Our combined approach leverages internationally available seismic datasets to resolve discrepancies in the published focal depth estimates (e.g., Girino et al.^33^; USGS^15^) and confirms a mid-crustal nucleation depth (21 ± 2 km) and right-lateral transpressional mechanism for the SJ2021 event. We suggest that the SJ2021 earthquake activated a NW-dipping, mid-crustal basement fault originally defined by microseismicity data beneath the Precordillera^27^, and posit that this and other basement structures may be kinematically linked to emergent faults associated with historically devastating shallow earthquakes in the Eastern Precordillera and Sierra de Pie de Palo.

Considered alongside crustal seismicity data and geological constraints, the SJ2021 provides opportunities to explore the mechanisms and interactions controlling deformation and stress conditions along variably oriented middle- and upper-crustal structures. We propose a model in which basement-involved deformation was largely driven by a mid-crustal stress guide parallel to the plate convergent direction and the reactivation of NW- and NE-striking, probably inherited faults; development of the fold-thrust belt was controlled by a separate stress guide modulated by topography body forces in the orogenic wedge; and partitioning of deformation along linked middle- and upper-crustal structures facilitated pure compression orthogonal to NE-striking emergent faults in the Eastern Precordillera and SdPP. Similar modes of stress transmission and middle- to upper-crustal structural connectivity have been proposed for the Sevier-Laramide domains of the North American Cordillera and may be a persistent mechanism of retroarc deformation in regions of elevated plate coupling and flat-slab subduction.

## Methods

### Earthquake relocation

The relocation procedure is based on seismic phase arrivals available from the ISC bulletin^13^, whose mission is to gather phase information from seismological agencies around the world. Although we do not have access to the seismic phases picked by INPRES, some of the data are publicly available from the ISC (ZON, RTLL and CFAA, see locations on Fig. 2a). Local phase information is critical to better constrain the hypocenter depth of the SJ2021.

First, seismic phases corresponding to stations for which the full waveform was available (Fig. S4) were manually re-picked. We did not observe significant differences between the ISC and our re-picked phases. We then calculated travel time tables for three different velocity models (Fig. S5): (1) A regional velocity model obtained from receiver function analysis^39^; (2) A velocity model obtained from the inversion of local P and S travel times^63^, and used in the analysis by Girino et al.^33^; (3) The CRUST1 model^64^, centered on the location of our study area (31.5$^{\circ }$S and 68.5$^{\circ }$W).

The hypocenter location of the SJ2021 was obtained using NonLinLoc^65^, a non-linear, grid-search algorithm based on the probabilistic reformulation of inverse problems^66^. Errors associated with observed arrivals and travel time calculations are assumed to be Gaussian, which allows the calculation of a maximum likelihood estimate of the origin time and location parameters. The resulting hypocenters are thus represented by an empirical density function, hence presenting a comprehensive uncertainty estimation (Fig. 2b).

Hypocenter solutions obtained using the different velocity models were similar. However, the solution obtained using Ammirati et al.^39^ velocity model was the most accurate in terms of residuals (RMS = 0.26 s) and hypocenter uncertainties (2.6 km, 3.2 km and 5.8 km in latitude, longitude and depth, respectively).

### Depth phase analysis

Two first arrival phases can be identified in the SJ2021 teleseismic waveforms, over a large variety of distances (60–180$^\circ$), with a consistent time delay of $\sim$8 s. These consistent time residuals of direct (P, PKIKP) and depth phases (pP, pPKIKP) are routinely used for teleseismic depth estimations^67^, and have been proven to estimate focal depths at least as accurately as regional catalogs^68^. Using the local 1D velocity model from Ammirati et al.^39^, an averaged P-wave crustal velocities of 5.73 km/s and the two-way travel time of $\sim$8s correspond to focal depths of $\sim$23 km.

The use of beamforming methods for teleseismic arrays can further enhance the signal quality by a factor of 4^68^. Hereby, the obtained waveforms from the individual sensors of the array are constructively stacked along the theoretical slowness vector of the arriving teleseismic wavefield. In this study, five different teleseismic arrays were used at epicentral distances between 69$^\circ$ and 120$^\circ$ (Fig. 3): Texas array (TXAR), Pinedale array (PDAR), Nevada Mina array (NVAR), Indian Mountain array (IMAR), and Alice Springs array (Alice). The beamforming parameters were estimated with the ray-tracer cake implemented in the Pyrocko package^69^. The beamformed waveforms of each array are then visually compared to a set of synthetic waveforms calculated for different source depths. The generation of synthetic waveforms is based on pre-calculated Green’s Functions^70^. The underlying velocity models are based on the AK135 global velocity model^71^.

We tested two sets of source depth ranges: (1) 15–30 km, with 1 km spacing; and (2) 23–25 km with a 0.1 km spacing. The beamformed waveforms are then visually aligned with the given synthetic waveforms as shown in Fig. 3. An attempted cross-correlation of the observed and synthetic waveforms was not successful. This may be due to a poor signal to noise ratio among the observed waveforms; the direct P phases in particular did not exhibit sufficient similarity. Interestingly, the overall higher amplitude depth phases also contained a longer duration of peak amplitudes. Whether this pattern is related to the moment duration, radiation pattern or a start and stop phase cannot be resolved with the present method.

### Regional moment tensor inversion

We use the open-source software Grond to derive an independent source solution^72–76^. The software employs a bootsrap-based Bayesian inversion approach with non-informative priors. Besides the efficiency and ability to properly sample model space uncertainties, this approach allows for the joint inversion of far-field seismic data and near-field static displacement observations, such as GNSS. Details of the inversion and how these diverse data are combined, are listed in the Supplementary material.

The source is constructed as a centroid moment tensor under a point source approximation. This assumption is valid with respect to consistently intermediate magnitude estimations in combination with the greater expected stress drop generated by interplate earthquakes^77,78^, reducing the overall slip area while maintaining the same seismic moment release. First tests that introduced a rectangular finite source model accounting for an extended source, or double double couple (double DC) sources targeted to capture the proposed start and stop phases observed in the beamforming of teleseismic depth phases, did not converge into stable solutions. Although the SJ2021 earthquake is best attributed to a tectonic origin, the source inversion is performed for the full moment tensor. Hereby, we allow the inversion to converge freely for the six moment tensor components and use the non-DC components to account for model errors stemming from the point source approximation and complex fault or rupture geometries^42^ or inaccurate velocity models. We solely relied on publicly available data for the full waveform moment tensor inversion. Waveforms were queried through the FDSN services of Geofon and USGS (networks used: Universidad de Chile, IPOC, Geoscope, IRIS/USGS) within a 1000 km search radius around the GEOFON earthquake location (Table S1, Fig. S4) and restricted to the availability of meta information necessary for the automatic restitution. An automated data quality check and manual revision removed several stations that were unusable for the inversion due to: (1) clipped recordings in the vicinity of the epicenter (e.g., station WA.ZON); (2) failed restitution; (3) alteration of the waveforms by local site effects (i.e., all stations located in or at the limit of the Santiago basin, Chile); or (4) insufficient signal to noise ratios to resolve the phases of interest at greater epicentral distances. The latter case was even applied to only one component for two stations (radial component of C1.BI02 and C1.MT04). Ultimately, waveform data from 35 stations was integrated into the moment tensor inversion (Fig. 2a). Preparation of the GNSS data required more sophisticated processing to derive the static displacement and associated uncertainties related to the SJ2021 event and is described in the following section.

The underlying velocity model for constructing the Green’s functions database is based on a combination of the local Crust1.0 model^64^ and the global AK135 model^71^. This integration allows for a spatial resolution of 1 km for the source region from 1 km down to 30 km depth and epicentral distance ranging from 10 to 1000 km. The same velocity model was used to derive the Green’s function database for static displacements modeling with a spatial resolution of 500 m. We first used the velocity model by Ammirati et al.^39^, completed by the AK135 model^71^, but the results were not fully satisfactory, which may stem from how this model is calibrated for the retroarc region of the Chilean-Argentine flat-slab segment and thus, does not account for the Chilean forearc velocity structure, where most of the stations used in the inversion are located.

### GNSS time series analysis

To assess crustal displacements around the SJ2021 epicenter, we analyzed the north, east, and up-position time series data from 4 GNSS stations within a $\sim$50 km range of the earthquake. These time series were computed and distributed by the Nevada Geodetic Laboratory (NGL) at the University of Nevada Reno^79^ thanks to the dissemination of GNSS station observation files by the Universidad Nacional de San Juan, the Complejo Astronómico El Leoncito, and the Instituto Geográfico Nacional. Each position time series was processed by the NGL using the GipsyX software^80^, with a single-station precise point positioning with carrier phase ambiguity resolution strategy based on Global Positioning System (GPS) observations only^81^. The products used in this study are the daily position estimates based on the final orbit and clock products provided by the Jet Propulsion Laboratory. We refer to Kreemer et al.^82^ for further information about GNSS data processing.

To estimate the co-seismic displacements caused by the SJ2021 with realistic uncertainties, we adjusted two complementary statistical models on each series: a trajectory model and a stochastic model. The trajectory model (Fig. S10) describes the deterministic effects identified in the position time series^83^, namely a linear trend, periodic oscillations, step discontinuities, outliers, and non-linear post-seismic deformations. The most frequently observed periodic oscillations, that is, the annual, biannual, and terannual signals, the 8 main GPS draconitic harmonics, and the 3 fortnightly signals, are considered^84,85^. For step discontinuities, we accounted for all instrumental changes or potential co-seismic displacements identified in the NGL’s master step file database (http://geodesy.unr.edu/NGLStationPages/steps.txt). A position estimate was considered an outlier if the estimate displayed a formal error over 10 mm or deviated from the monthly medians by over 6 times the median absolute deviation. Lastly, when required, we described the non-linear post-seismic displacements due to the 2010 (Mw 8.9) Maule and 2015 (Mw 8.3) Illapel earthquakes (Both are megathrust earthquakes occured on the Chilean margin), using exponential relaxation functions^86^.

The stochastic model describes the stochastic variations in the position time series, often called “noise”. This model consisted of a linear combination of a white noise process and a flicker noise process to account for time correlations and obtain realistic displacement uncertainties^87^. The unknown amplitudes of these stochastic processes were estimated from observations using the Least-Square Variance Component Estimation method^88^. Once these stochastic process amplitudes were estimated, the unknown parameters of the trajectory model and their uncertainties were calculated using the best linear unbiased estimator^88^, yielding the coseismic displacement (Fig. 2b, Table S2).

## Supplementary Information

Supplementary Information.

## Data Availability

The waveforms used in this study can be downloaded from the IRIS Data management center (IRISDMC) at http://service.iris.edu/fdsnws/dataselect/1/ or the GEOFON EIDA node at http://eida.gfz-potsdam.de/webdc3/. Network codes are C, C1, G, IU and WA. GNSS time series data are available from the Nevada Geodetic Laboratory at http://geodesy.unr.edu/NGLStationPages/GlobalStationList. Station codes are CSJ1, CSLO, OAFA and USNJ. All websites were last accessed in October 2022.

## References

[1] Fitch TJ (1972). Plate convergence, transcurrent faults, and internal deformation adjacent to southeast Asia and the western Pacific. J. Geophys. Res..

[2] Dickinson WR, Snyder WS, Matthews V (1978). Plate Tectonics of the Laramide Orogeny.

[3] Jordan TE, Allmendinger RW (1986). The Sierras Pampeanas of Argentina; a modern analogue of Rocky Mountain foreland deformation. Am. J. Sci..

[4] Cahill T, Isacks BL (1992). Seismicity and shape of the subducted Nazca plate. J. Geophys. Res. Solid Earth.

[5] Erslev, E. A. & Koenig, N. V. Three-Dimensional Kinematics of Laramide, Basement-Involved Rocky Mountain Deformation, USA: Insights from Minor Faults and GIS-Enhanced Structure Maps (2009).

[6] Ramos V, Cristallini E, Pérez D (2002). 7KH 3DPSHDQ flDW slab of the Central Andes. J. S. Am. Earth Sci..

[7] Varga RJ (1993). Rocky mountain foreland uplifts: products of a rotating stress field or strain partitioning?. Geology.

[8] Erslev, E. A., Schmidt, C. & Chase, R. Thrusts, back-thrusts and detachment of rocky mountain foreland arches. Special Papers-Geological Society of America 339–339 ( 1993).

[9] Siame L (2002). Seismic hazard reappraisal from combined structural geology, geomorphology and cosmic ray exposure dating analyses: the Eastern Precordillera thrust system (NW Argentina). Geophys. J. Int..

[10] Siame L, Bellier O, Sébrier M, Araujo M (2005). Deformation partitioning in flat subduction setting: case of the Andean foreland of western Argentina (28$${\hat{\,}}$$os 33$${\hat{\,}}$$os). Tectonics.

[11] Vergés, J. et al. Crustal wedging triggering recent deformation in the Andean thrust front between 31 s and 33 s: Sierras Pampeanas-Precordillera interaction. *J. Geophys. Res. Solid Earth* **112** (2007).

[12] Yonkee WA, Weil AB (2015). Tectonic evolution of the Sevier and Laramide belts within the North American Cordillera orogenic system. Earth Sci. Rev..

[13] International Seismological Centre. On-line Bulletin (2022). http://www.isc.ac.uk/iscbulletin/search/.

[14] Instituto Nacional de Prevención Sísmica, terremotos históricos ocurridos en la república Argentina (2022). http://contenidos.inpres.gob.ar/sismologia/historicos.

[15] United States Geological Survey-Earthquake Hazard Program, m 6.4–26 km sw of pocito, Argentina (2022). https://earthquake.usgs.gov/earthquakes/eventpage/us7000d18q/executive.

[16] Hayes GP (2018). Slab2, a comprehensive subduction zone geometry model. Science.

[17] Ramos, V. A. The tectonics of the Central Andes; 30 to 33 S latitude (1988).

[18] Siame LL (2015). Active basement uplift of Sierra Pie de Palo (Northwestern Argentina): rates and inception from10be cosmogenic nuclide concentrations. Tectonics.

[19] DeCelles P, Graham S (2015). Cyclical processes in the North American Cordilleran orogenic system. Geology.

[20] Von Gosen W (1992). Structural evolution of the argentine precordillera: the Río San Juan section. J. Struct. Geol..

[21] Zapata TR, Allmendinger RW (1996). Growth stratal records of instantaneous and progressive limb rotation in the Precordillera thrust belt and Bermejo basin, Argentina. Tectonics.

[22] Snyder D, Ramos V, Allmendinger R (1990). Thick-skinned deformation observed on deep seismic reflection profiles in western Argentina. Tectonics.

[23] Alvarado P, Beck S (2006). Source characterization of the San Juan (Argentina) crustal earthquakes of 15 January 1944 (MW 7.0) and 11 June 1952 (MW 6.8). Earth Planet. Sci. Lett..

[24] Meigs A, Krugh WC, Schiffman C, Vergés J, Ramos VA (2006). Refolding of thin-skinned thrust sheets by active basement-involved thrust faults in the eastern precordillera of western Argentina. Rev. Asoc. Geol. Argent..

[25] Bellahsen N, Sébrier M, Siame L (2016). Crustal shortening at the Sierra Pie de Palo (Sierras Pampeanas, Argentina): near-surface basement folding and thrusting. Geol. Mag..

[26] Regnier M (1992). Seismotectonics of Sierra Pie de Palo, a basement block uplift in the Andean foreland of Argentina. Bull. Seismol. Soc. Am..

[27] Smalley R (1993). Basement seismicity beneath the Andean Precordillera thin-skinned thrust belt and implications for crustal and lithospheric behavior. Tectonics.

[28] Langer C, Hartzell S (1996). Rupture distribution of the 1977 western Argentina earthquake. Phys. Earth Planet. Inter..

[29] Alvarado P, Beck S, Zandt G, Araujo M, Triep E (2005). Crustal deformation in the south-central Andes backarc terranes as viewed from regional broad-band seismic waveform modelling. Geophys. J. Int..

[30] Brooks, B. A. et al. Crustal motion in the Southern Andes (26$$^{\circ }$$–36$$^{\circ }$$ s): do the Andes behave like a microplate? *Geochem. Geophys. Geosyst.* **4** (2003).

[31] Heidbach O (2018). The World Stress Map database release 2016: crustal stress pattern across scales. Tectonophysics.

[32] Venerdini A (2020). Crustal seismicity in the Andean Precordillera of Argentina using seismic broadband data. Tectonophysics.

[33] Girino GS (2021). El terremoto (Mw 6.4) superficial del 18 de enero de 2021 del Cordón de las Osamentas, Precordillera Central, San Juan, Argentina. Rev. Asoc. Geol. Argent..

[34] Costa, C. Strong quake rattles San Juan (2021). 10.32858/temblor.154.

[35] Chatelain J-L, Roecker S, Hatzfeld D, Molnar P (1980). Microearthquake seismicity and fault plane solutions in the Hindu Kush region and their tectonic implications. J. Geophys. Res. Solid Earth.

[36] Kissling E (1988). Geotomography with local earthquake data. Rev. Geophys..

[37] Gomberg JS, Shedlock KM, Roecker SW (1990). The effect of s-wave arrival times on the accuracy of hypocenter estimation. Bull. Seismol. Soc. Am..

[38] Barrientos S (2018). The seismic network of Chile. Seismol. Res. Lett..

[39] Ammirati J-B, Alvarado P, Beck S (2015). A lithospheric velocity model for the flat slab region of Argentina from joint inversion of Rayleigh wave phase velocity dispersion and teleseismic receiver functions. Geophys. J. Int..

[40] Zeckra, M., Gobron, K., Ammirati, J.-B. & Mackaman-Lofland, C. Moment tensor solution for the 2021 San Juan M6.4 earthquake based on joint inversion of full waveforms and GNSS static displacements, ISC seismological dataset repository (2022). 10.31905/K3J1V6RM.

[41] Frohlich C (1995). Characteristics of well-determined non-double-couple earthquakes in the Harvard CMT catalog. Phys. Earth Planet. Inter..

[42] Adamová P, Šílenỳ J (2010). Non-double-couple earthquake mechanism as an artifact of the point-source approach applied to a finite-extent focus. Bull. Seismol. Soc. Am..

[43] Chiu J, Steiner G, Smalley R, Johnston A (1991). Panda: a simple, portable seismic array for local-to regional-scale seismic experiments. Bull. Seismol. Soc. Am..

[44] Beck, S. & Zandt, G. Lithospheric structure and deformation of the flat slab region of Argentina. International Federation of Digital Seismograph Networks. Dataset/Seismic Network (2007).

[45] Ammirati J-B (2016). High-resolution images above the Pampean flat slab of Argentina (31–32 s) from local receiver functions: implications on regional tectonics. Earth Planet. Sci. Lett..

[46] Smalley R, Isacks BL (1990). Seismotectonics of thin-and thick-skinned deformation in the Andean foreland from local network data: evidence for a seismogenic lower crust. J. Geophys. Res. Solid Earth.

[47] Perucca L, Paredes J (2002). Peligro sísmico en el departamento Albardón y su relación con el área de fallamiento La Laja, provincia de San Juan. Rev. Assoc. Geol. Argent..

[48] Chinn DS, Isacks BL (1983). Accurate source depths and focal mechanisms of shallow earthquakes in western South America and in the New Hebrides island arc. Tectonics.

[49] Kadinsky-Cade K, Reilinger R, Isacks B (1985). Surface deformation associated with the November 23, 1977, Caucete, Argentina, earthquake sequence. J. Geophys. Res. Solid Earth.

[50] Meigs, A. J. & Nabelek, J. Crustal-scale pure shear foreland deformation of western Argentina. *Geophys. Res. Lett.* **37** (2010).

[51] Gries R (1983). Oil and gas prospecting beneath Precambrian of foreland thrust plates in Rocky mountains. AAPG Bull..

[52] Molzer PC, Erslev EA (1995). Oblique convergence during northeast-southwest laramide compression along the east-west Owl Creek and Casper Mountain arches, central Wyoming. AAPG Bull..

[53] Stone DS (1986). Seismic and Borehole Evidence for Important pre-Laramide Faulting Along the Axial Arch in Northwest Colorado.

[54] Marshak S, Karlstrom K, Timmons JM (2000). Inversion of Proterozoic extensional faults: an explanation for the pattern of Laramide and Ancestral Rockies intracratonic deformation, United States. Geology.

[55] Müller, R. D., Sdrolias, M., Gaina, C. & Roest, W. R. Age, spreading rates, and spreading asymmetry of the world’s ocean crust. *Geochem. Geophys. Geosyst.* **9** (2008).

[56] Maloney KT, Clarke GL, Klepeis KA, Quevedo L (2013). The late Jurassic to present evolution of the Andean margin: drivers and the geological record. Tectonics.

[57] Elliott D (1976). A discussion on natural strain and geological structure-the energy balance and deformation mechanisms of thrust sheets. Philos. Trans. R. Soc. Lond. Ser. A Math. Phys. Sci..

[58] Davis D, Suppe J, Dahlen F (1983). Mechanics of fold-and-thrust belts and accretionary wedges. J. Geophys. Res. Solid Earth.

[59] Siame LL (1997). Cosmogenic dating ranging from 20 to 700 ka of a series of alluvial fan surfaces affected by the El Tigre fault, Argentina. Geology.

[60] Alvarez-Marron J (2006). Neogene structures overprinting palaeozoic thrust systems in the Andean Precordillera at 30$$^{circ}$$ s latitude. J. Geol. Soc..

[61] Otamendi JE (2009). Geology and petrology of a deep crustal zone from the Famatinian paleo-arc, Sierras de Valle Fértil and La Huerta, San Juan, Argentina. J. S. Am. Earth Sci..

[62] Bastías HE, Bastías JA (1987). Análisis de desplazamientos y velocidades en el área diferencial Precordillera, provincia de San Juan. Rev. Asoc. Geol. Argent..

[63] Venerdini A, Sánchez G, Alvarado P, Bilbao I, Ammirati J-B (2016). Nuevas determinaciones de velocidades de ondas p y ondas s para la corteza sísmica del terreno cuyania en el retroarco andino. Rev. Mex. Ciencias Geol..

[64] Laske, G., Masters, G., Ma, Z. & Pasyanos, M. Update on crust1. 0–a 1-degree global model of earth’s crust, in *Geophysical Research Abstracts*, vol. 15, 2658 (EGU General Assembly Vienna, 2013).

[65] Lomax, A., Virieux, J., Volant, P. & Berge-Thierry, C. Probabilistic earthquake location in 3d and layered models, in *Advances in Seismic Event Location* 101–134 (Springer, 2000).

[66] Tarantola A, Valette B (1982). Generalized nonlinear inverse problems solved using the least squares criterion. Rev. Geophys..

[67] Engdahl ER, van der Hilst R, Buland R (1998). Global teleseismic earthquake relocation with improved travel times and procedures for depth determination. Bull. Seismol. Soc. Am..

[68] Woodgold CR (1999). Wide-aperture beamforming of depth phases by timescale contraction. Bull. Seismol. Soc. Am..

[69] Heimann, S. et al. Pyrocko-An open-source seismology toolbox and library (2017).

[70] Heimann S (2019). A python framework for efficient use of pre-computed green’s functions in seismological and other physical forward and inverse source problems. Solid Earth.

[71] Kennett, B. *Seismological Tables: ak135* 1–289 (Research School of Earth Sciences, Australian National University Canberra, 2005).

[72] Heimann, S. et al. Grond: a probabilistic earthquake source inversion framework (2018).

[73] Dahm T (2018). Seismicity in the block mountains between Halle and Leipzig, Central Germany: centroid moment tensors, ground motion simulation, and felt intensities of two m$$\approx $$3 earthquakes in 2015 and 2017. J. Seismol..

[74] Kühn D, Heimann S, Isken MP, Ruigrok E, Dost B (2020). Probabilistic moment tensor inversion for hydrocarbon-induced seismicity in the Groningen gas field, the Netherlands, part 1: Testing. Bull. Seismol. Soc. Am..

[75] Petersen GM (2021). Regional centroid moment tensor inversion of small to moderate earthquakes in the Alps using the dense Alparray seismic network: challenges and seismotectonic insights. Solid Earth.

[76] Valenzuela-Malebrán C (2022). Source mechanisms and rupture processes of the Jujuy seismic nest, Chile-Argentina border. J. S. Am. Earth Sci..

[77] Scholz CH, Aviles C, Wesnousky SG (1986). Scaling differences between large interplate and intraplate earthquakes. Bull. Seismol. Soc. Am..

[78] Kato, N. A possible explanation for difference in stress drop between intraplate and interplate earthquakes. *Geophys. Res. Lett.* **36** (2009).

[79] Blewitt G, Hammond WC, Kreemer C (2018). Harnessing the GPS data explosion for interdisciplinary science. Eos.

[80] Bertiger W (2020). GipsyX/RTGx, a new tool set for space geodetic operations and research. Adv. Space Res..

[81] Zumberge J, Heflin M, Jefferson D, Watkins M, Webb F (1997). Precise point positioning for the efficient and robust analysis of GPS data from large networks. J. Geophys. Res. Solid Earth.

[82] Kreemer C, Blewitt G, Davis PM (2020). Geodetic evidence for a buoyant mantle plume beneath the Eifel volcanic area, NW Europe. Geophys. J. Int..

[83] Bevis M, Brown A (2014). Trajectory models and reference frames for crustal motion geodesy. J. Geod..

[84] Ray J, Altamimi Z, Collilieux X, van Dam T (2008). Anomalous harmonics in the spectra of GPS position estimates. GPS Solut..

[85] Amiri-Simkooei A (2013). On the nature of GPS draconitic year periodic pattern in multivariate position time series. J. Geophys. Res. Solid Earth.

[86] Savage J, Prescott W (1978). Asthenosphere readjustment and the earthquake cycle. J. Geophys. Res. Solid Earth.

[87] Zhang J (1997). Southern California permanent GPS geodetic array: error analysis of daily position estimates and site velocities. J. Geophys. Res. Solid Earth.

[88] Teunissen PJ, Amiri-Simkooei AR (2008). Least-squares variance component estimation. J. Geodesy.

