# Supplementary material for: Stress transmission along mid-crustal faults highlighted by the 2021 Mw 6.5 San Juan (Argentina) earthquake
Source: Sci Rep. 2022 Oct 26;12:17939. doi: 10.1038/s41598-022-22752-6 (PMC9606362; doi:10.1038/s41598-022-22752-6)
Supplement: Supplementary file 1 — Supplementary Information. [file 41598_2022_22752_MOESM1_ESM.pdf]

Supplementary information for **"Stress transmission along mid-crustal faults highlighted by the 2021 Mw 6.5 San Juan (Argentina) earthquake"**

Jean-Baptiste Ammirati[1,*], Chelsea Mackaman-Lofland[2,3] , Martin Zeckra[4] and Kevin Gobron [4,5]

[1]Departamento de Geología, Universidad de Chile, Plaza Ercilla 803, 8370450 Santiago, Chile

[2] Department of Geosciences, University of Connecticut, Beach Hall, 354 Mansfield Rd #207, Storrs, CT 06269, USA

[3] Department of Earth and Environmental Sciences, Denison University, 100 West College St, Granville, Ohio 43023, USA

[4] Royal Observatory of Belgium, Avenue Circulaire 3, 1180 Uccle, Belgium

[5] Université de Paris Cité, Institut de physique du globe de Paris, CNRS, IGN, F-75005 Paris, France

*Corresponding author now at University of Lausanne, Institute of Earth Sciences, Lausanne Switzerland, jean-baptiste.ammirati@unil.ch

Introduction

The January 19th San Juan (Mw=6.5) earthquake was recorded by many seismic networks around the globe. It has been located in the Eastern Precordillera of San Juan (western Argentina) about 50 km southwest of the city of San Juan and ~114 km north of the city of Mendoza, the two main urban concentrations in this area.

Table 1 summarizes the solutions obtained from both close and distant observations by local and international agencies. Table 2 reports the static displacement (and associated

uncertainties) linked to the 2021 San Juan earthquake (SJ2021), recorded by 4 nearby GNSS stations. Figure S1 shows the LOS displacements computed from InSAR interferograms.

Figure S2 presents the results of simple synthetic static displacement simulation for an earthquake characterized by a magnitude Mw=6.5 and two different focal depths, at 5 and 20 km, respectively.

Figure S3 is a record section showing the seismograms corresponding to the 2021 San Juan Earthquake recorded by one station in Argentina (ZON, http://service.iris.edu/fdsnws/event/1/) and stations from the Chilean seismic network (http://sismologia.cl/). The saturation of the seismograms at local and regional distances might be one of the reasons explaining the differences in focal depth estimations between local and distant (teleseismic) observations. Figure S4 shows a comparison of three different velocity models used to relocate the SJ2021 (see main text). Figure S5 to S8 shows the GNSS displacement and fitted trajectory model around the date of the SJ2021. Figure S9 shows the same information as Fig. S5-S8, except for a longer period of observation, accounting for several megathrust earthquakes that occurred along the Chilean Margin in 2010 (Maule, Mw=8.9) and 2014 (Illapel, Mw=8.3).

Finally we give more information about the methodology employed for our full moment tensor inversion for which the complete report is publically available at https://doi.org/10.31905/K3J1V6RM (last accessed October 2022).

Table S1: SJ2021 source parameters from different agencies. INPRES: Instituto Nacional de Prevención sísmica (Argentina); CSN: Centro Sismológico Nacional (Chile); USGS: US Geological Survey; GCMT: Global Centroid Moment Tensor, Lamont-Doherty Earth Observatory (LDEO) of Columbia University; GEOSCOPE: French Global Network of broadband seismic stations. (*) The alternate depth of 5.6 km and focal mechanism solution comes from Girino et al. (2021). (**) The CSN Focal mechanism was obtained by unsupervised regional waveform moment tensor inversion (Delouis, 2014; Derode et al., 2019). Lon: Longitude; Lat: Latitude; Mag.: Magnitude. Latitude, longitude, strike dip and rake are in degrees.

Agency	Lat	Lon	depth (km)	Mag type	Mag	Str1	Dip1	Rake1	str2	dip2	Rake2
INPRES*	-31.850	-68.936	8 (5.6)	M_L	6.4	219	78	172	311	82	12
CSN**	-31.833	-69.031	10	Mww	6.4	217	61	139	330	55	36
USGS	-31.833	-68.799	20.8	Mww	6.4	227	57	175	320	86	33
GCMT	-31.85	-68.86	25.8	Mw	6.5	223	61	164	321	76	30
GEOSCOPE	-31.837	-68.813	21	Mw	6.5	215	51	152	323	69	43
GEOFON	-31.807	-68.736	14	Mw	6.4	224	72	169	318	79	17
This Work	-31.812	-68.935	21.4	Mw	6.5	223	66	163	320	75	25

Table S2: Estimated North, East and Up displacements caused by the San Juan earthquake for the CSLO, UNSJ, CSJ1 and OAFA stations. STD: Standard deviation.

Name	Lon [°]	Lat [°]	North [mm]	East [mm]	Up [mm]	STD North [mm]	STD East [mm]	STD Up [mm]
CSLO	-69.302	-31.785	-2.31	17.49	-6.17	0.66	0.71	1.93
UNSJ	-68.577	-31.785	2.55	-0.53	1.53	0.70	0.65	1.88
CSJ1	-68.427	-31.981	1.64	-7.01	-1.86	0.69	0.62	2.30
OAFA	-68.623	-31.509	4.95	1.92	3.96	0.77	0.87	2.42

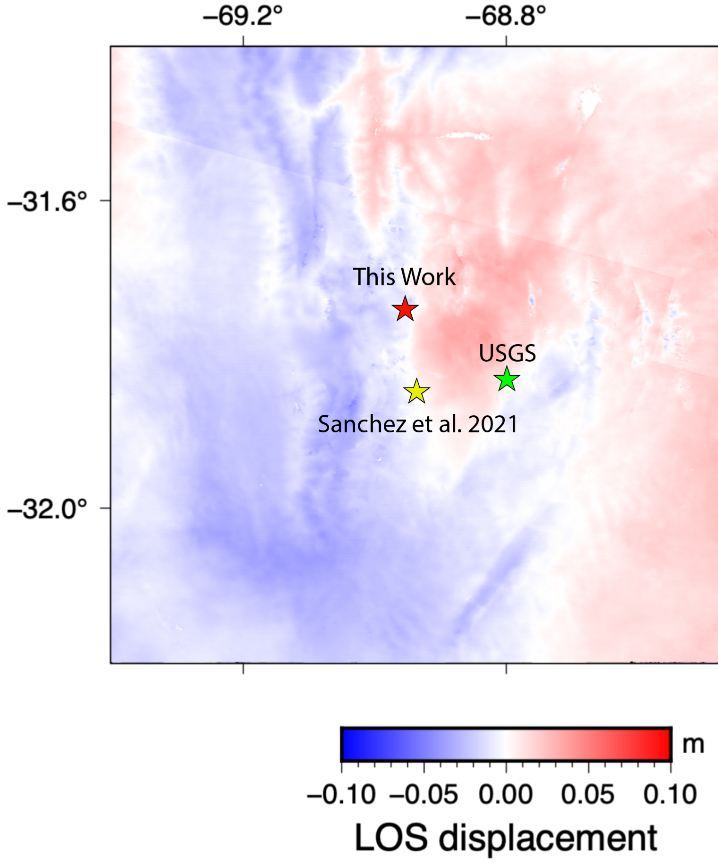

Figure S1: Map centered on our study region showing LOS displacement during the 2021 San Juan Earthquake from InSAR images (Geohazard, 2022). Note how the displacements are mostly inferior to 5 cm and reveal strong topographic effects. We do not not discard some displacement related to the Earthquake (observed in GNSS times series, see main text) but the values would be mostly inferior to ~2 cm. Because of its magnitude (Mw6.5), this observation suggests a deep crustal character of the SJ2021 (~20 km). A shallower focal depth would have likely generated displacement larger than ~10 cm and would have been visible in the interferograms (also see Fig. S3). This map was created using the GMT package (V.6.0.0, https://www.generic-mapping-tools.org/).

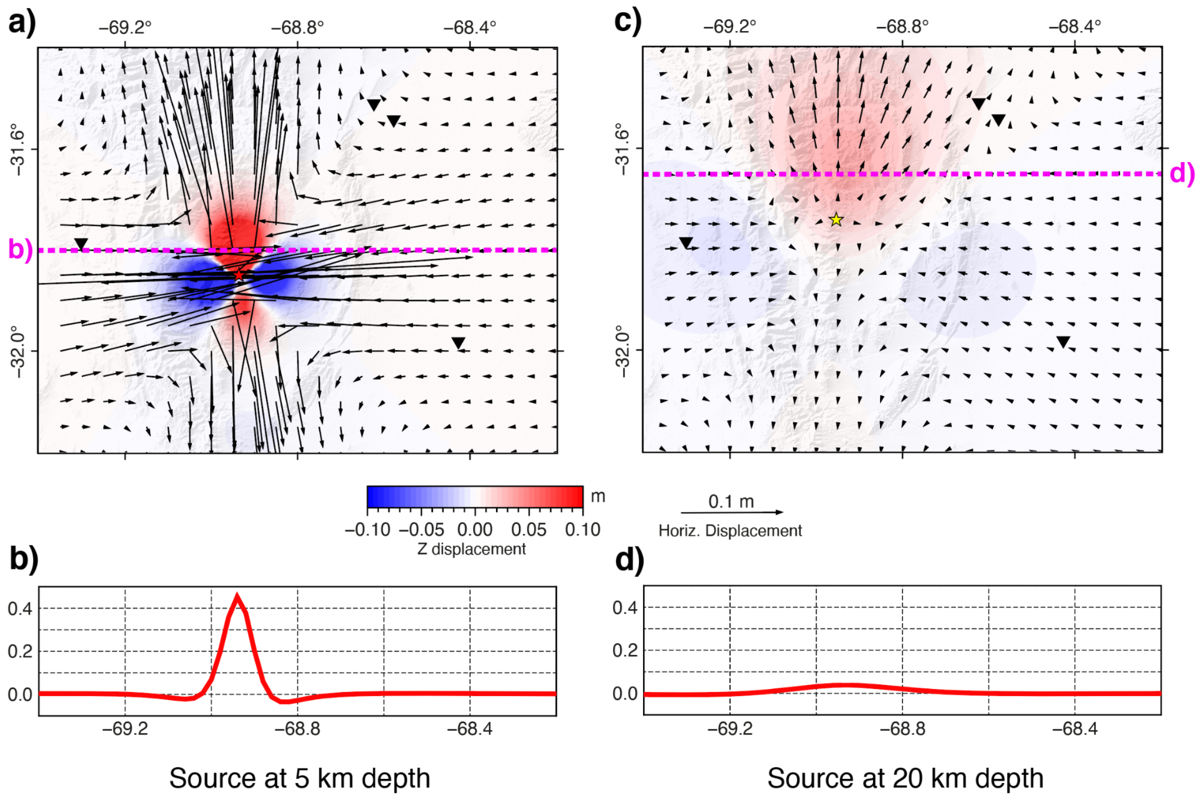

Figure S2: Horizontal and vertical synthetic static displacement calculated for source depths of 5 and 20 km (Hermann, 2013). a) The Mw 6.5 point-source is located at 5 km depth, similar to Girino et al. (2021) solution for the SJ2021. The red star marks the epicenter of the SJ2021 according to Girino et al (2021). b) Cross section along latitude 31.8ºS, showing the corresponding vertical static displacement amplitude. Note that, in this case the vertical static displacement reaches values larger than 40 cm. c) similar to a) but with a point source located at 20 km depth. The yellow star shows d) Cross section along latitude 31.65ºS showing the corresponding vertical static displacement amplitude. In this case, the maximum amplitude is about 0.03 m. Also, the synthetic static displacement values appear closer to the coseismic displacement recorded by nearby GNSS stations (black inverted triangles). We used Girino et al. (2021) and the USGS focal mechanisms solution to run these simulations.

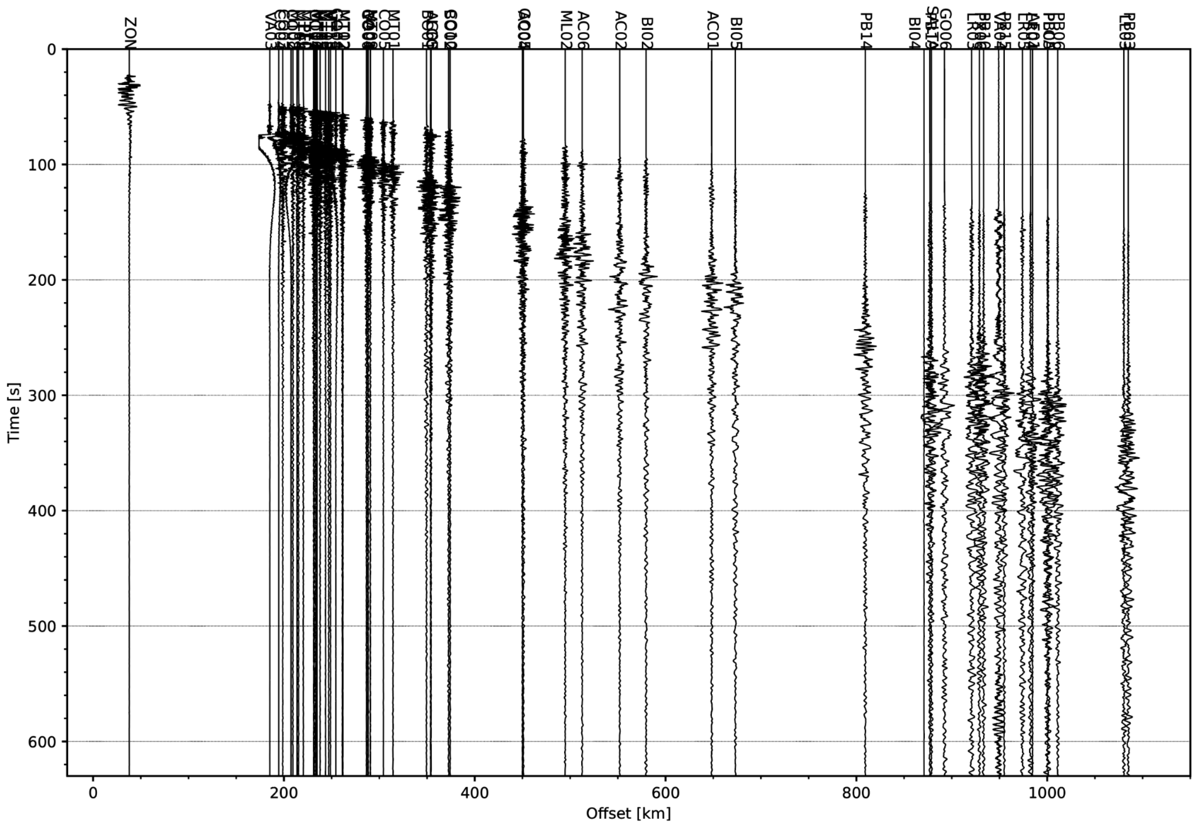

Figure S3: Record section (time versus distance) showing the vertical component of local and regional unfiltered waveforms corresponding to the SJ2021 event. Note the waveform saturation at epicentral distances up to ~300 km.

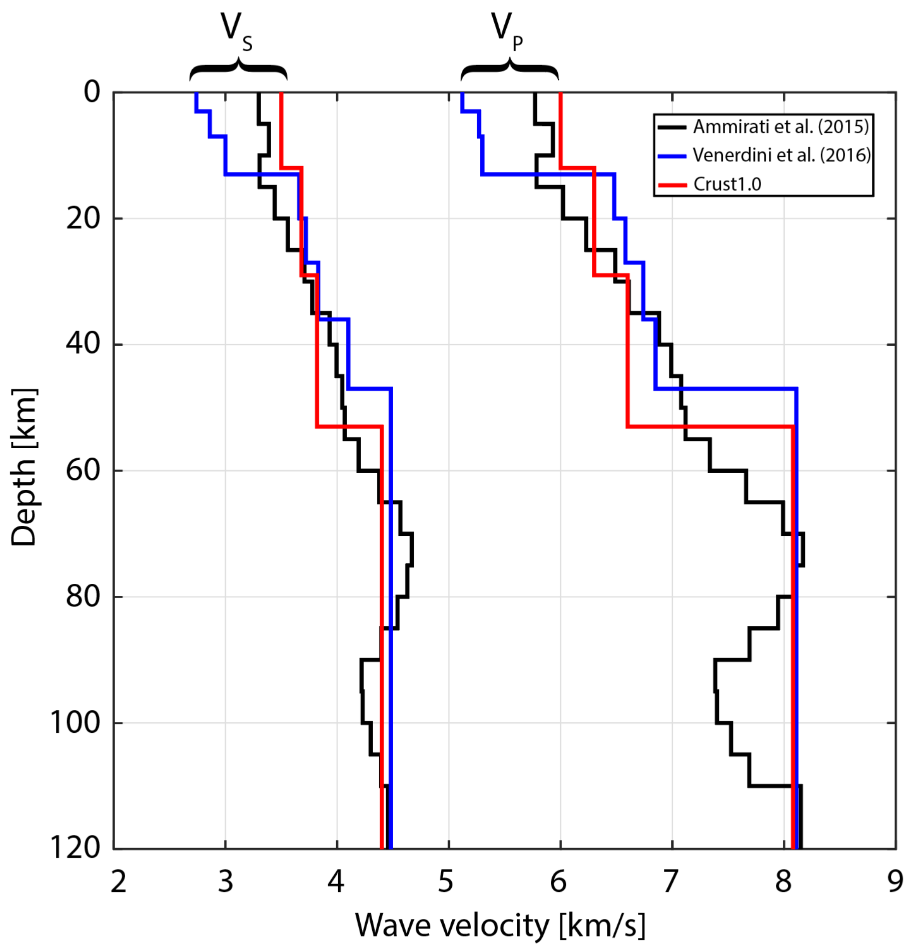

Figure S4: A comparison of the different velocity models used to relocate the SJ2021 (Ammirati et al. 2015; Venerdini et al., 2016; Laske et al. 2013)

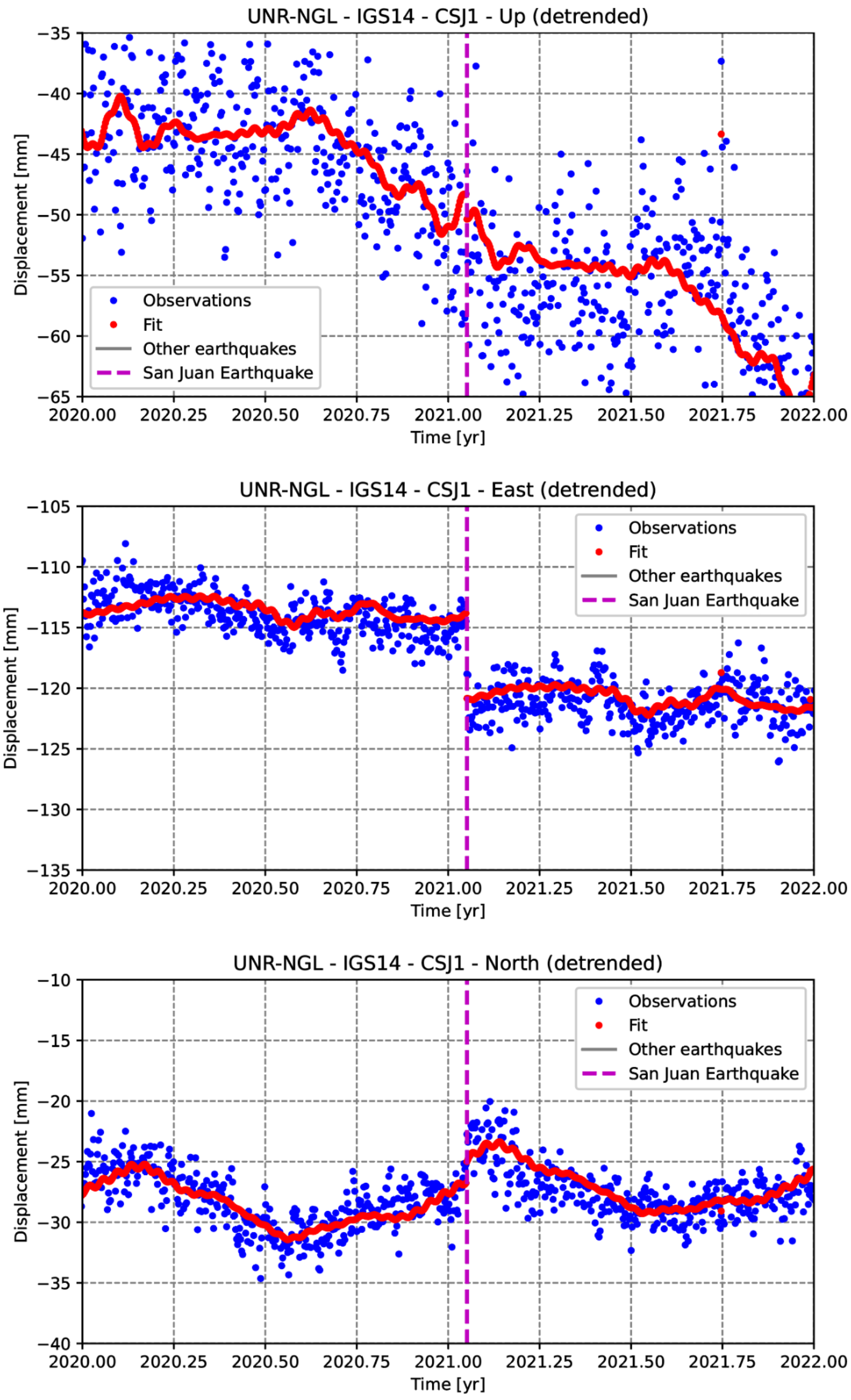

Figure S5: Multi-component GNSS displacement (blue dots) and trajectory model (red dots) observed at station CSJ1 for years 2020 and 2021 (see main text for more information).

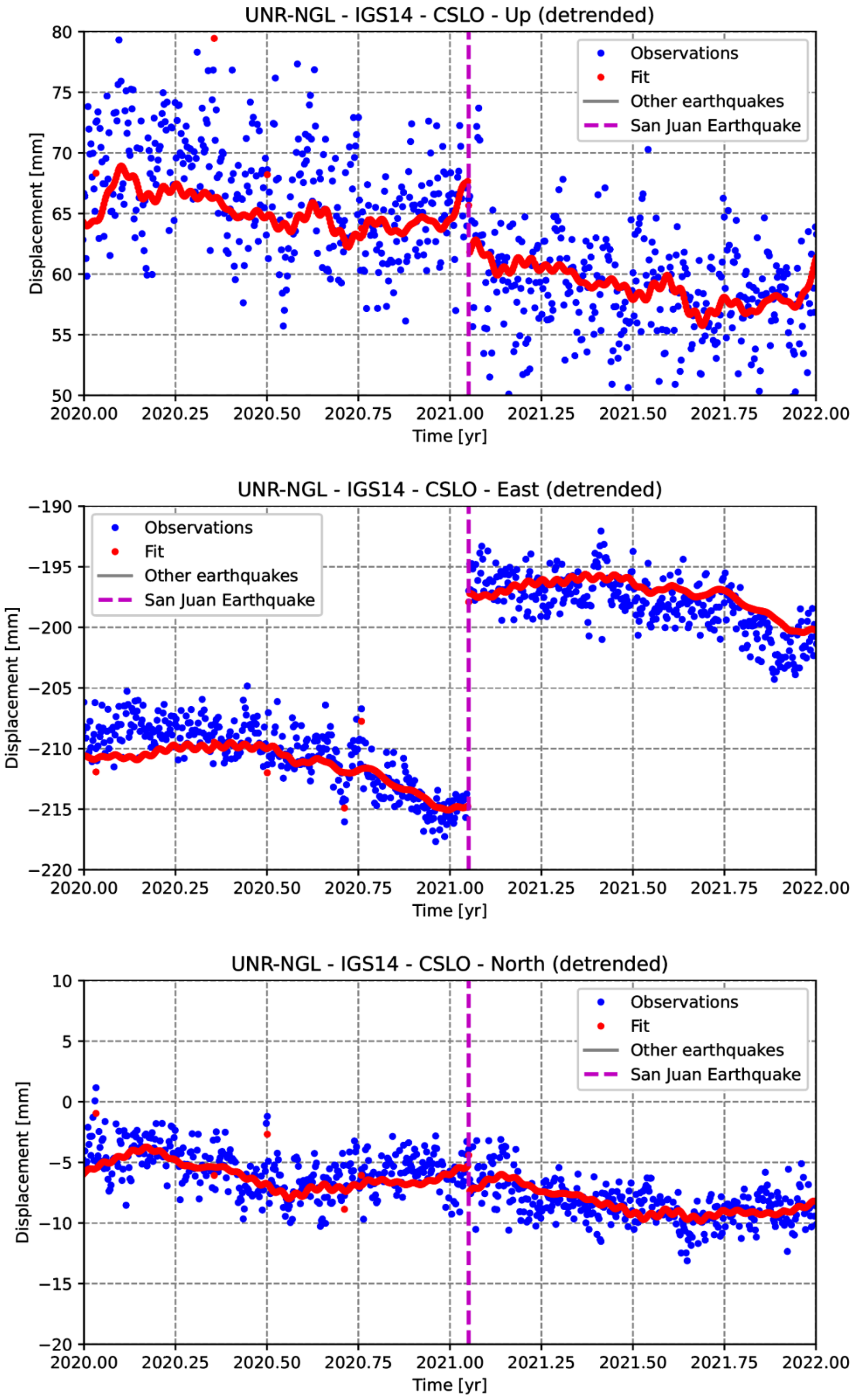

Figure S6: Multi-component GNSS displacement (blue dots) and trajectory model (red dots) observed at station CSLO for years 2020 and 2021 (see main text for more information).

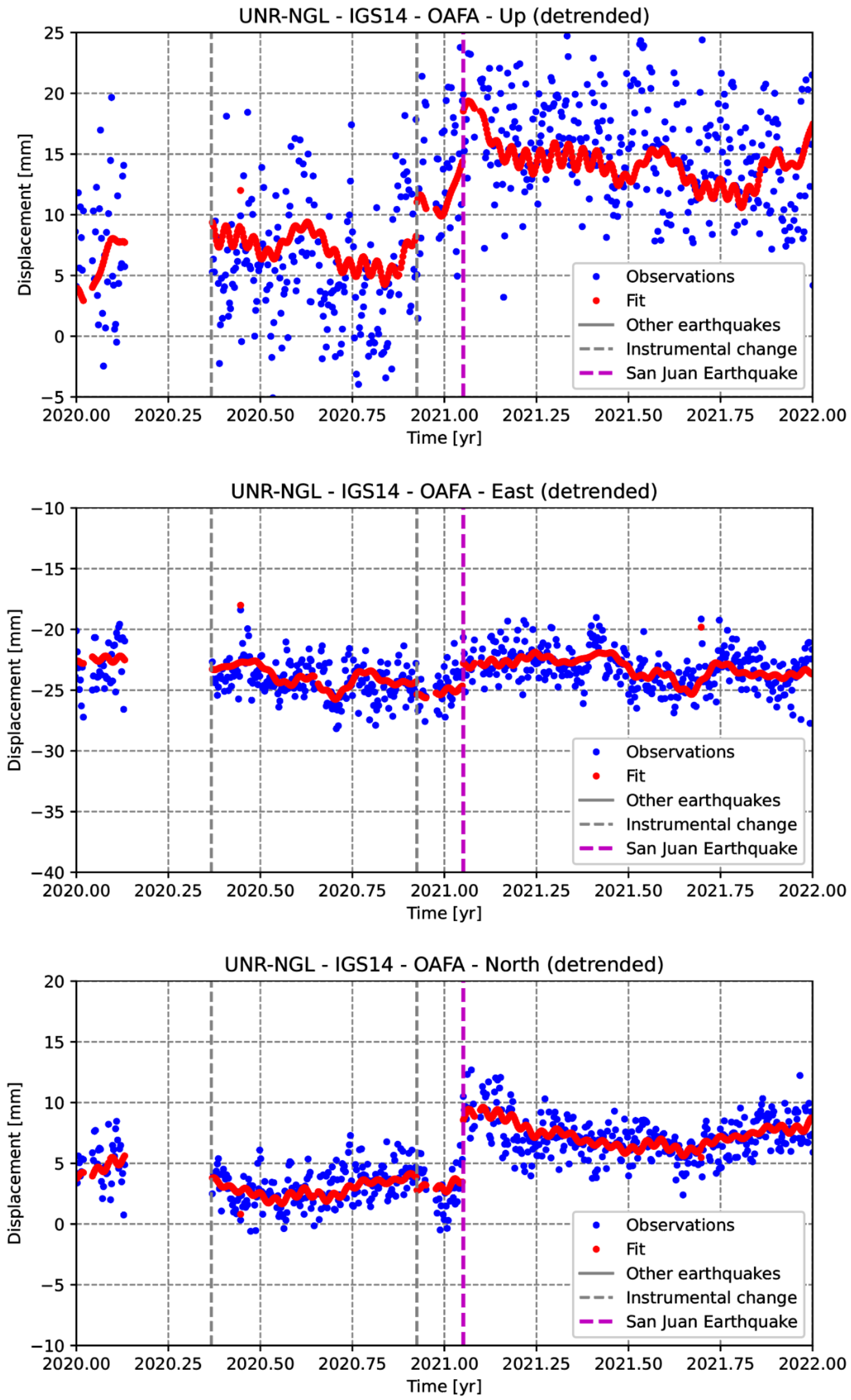

Figure S7: Multi-component GNSS displacement (blue dots) and trajectory model (red dots) observed at station OAFA for years 2020 and 2021 (see main text for more information).

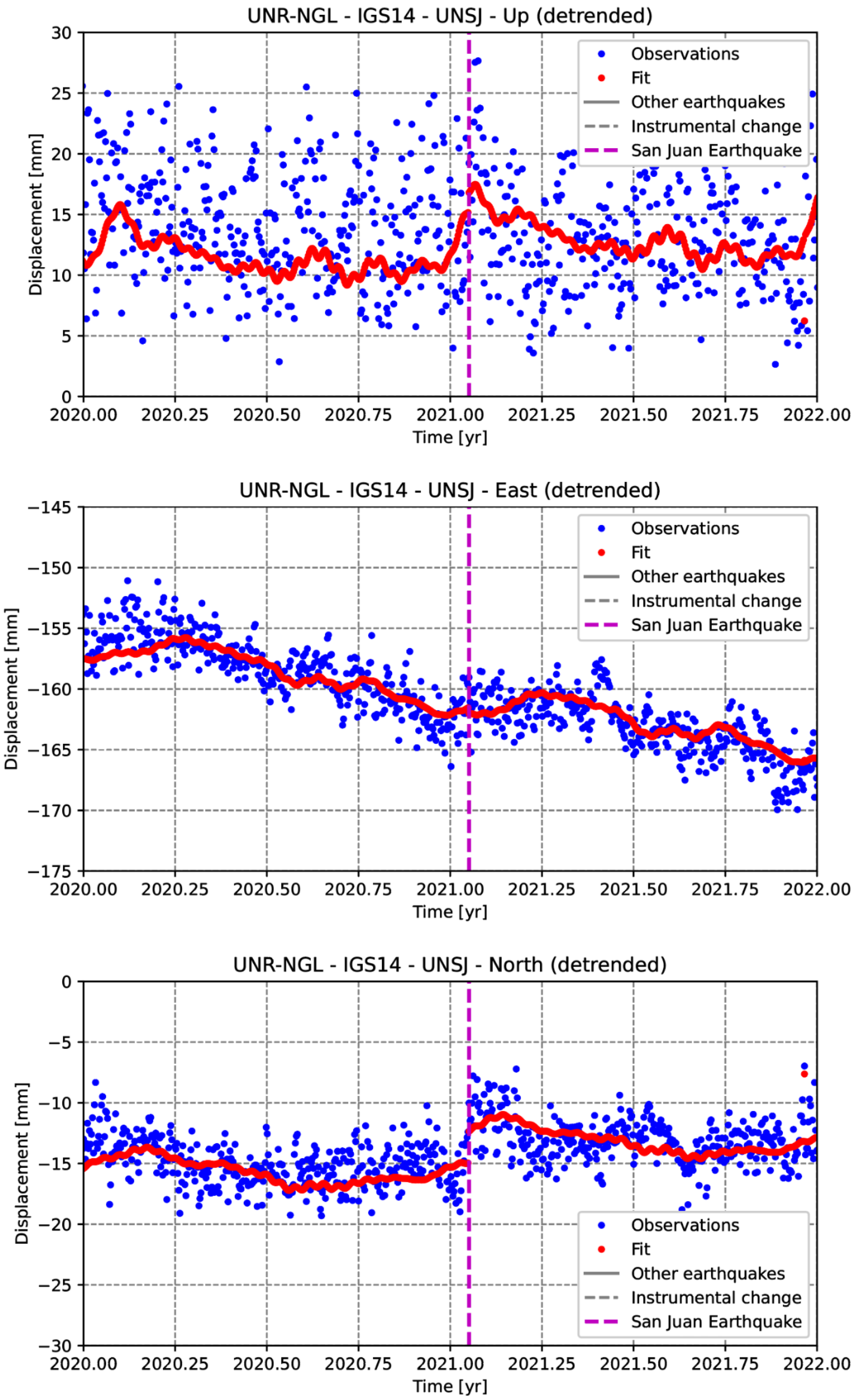

Figure S8: Multi-component GNSS displacement (blue dots) and trajectory model (red dots) observed at station UNSJ for years 2020 and 2021 (see main text for more information).

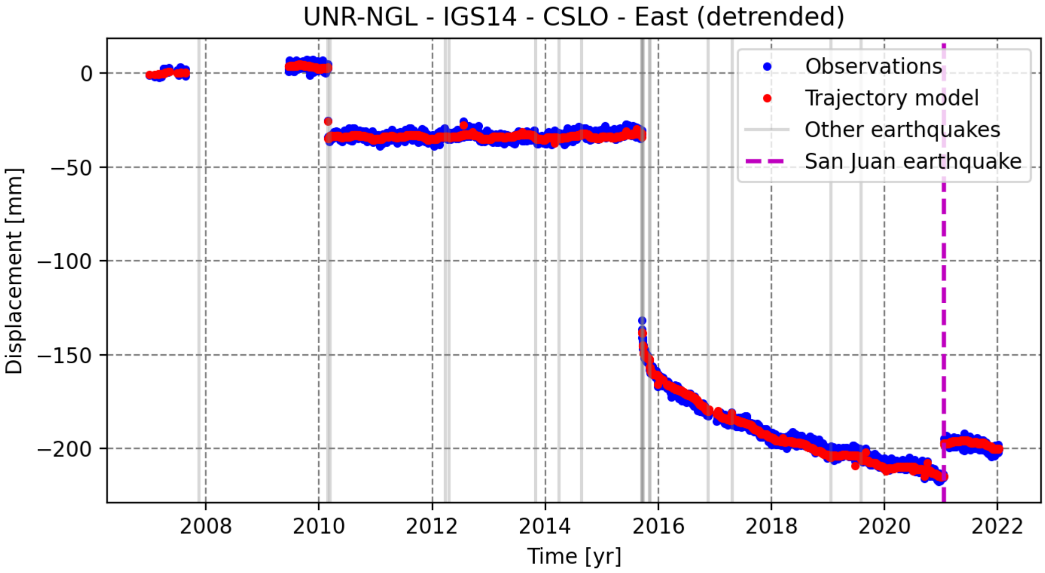

Figure S9: An example of the adjusted trajectory model, for the East coordinate of the CSLO station. A 17 mm displacement is visible at the date of the San Juan earthquake.

Bayesian joint-inversion

Strictly speaking, Grond is not applying an inversion in a mathematical definition (Kühn et al., 2020). Instead, a directed search algorithm is sampling the predefined solution space describing six independent moment tensor components, location and source time from which the objective function (or misfit function) is minimized as the misfit between observed and forward modeled, synthetic data. Efficient forward modeling is achieved through precomputed Green's function databases (Heimann et al., 2019) in a similar fashion as it was used during the beamforming of teleseismic depth phases. Synthetic static displacements for the integration of GNSS data used Green's functions that were calculated with the *PSGRN/PSCMP* backend (Wang et al., 2003, Wang 2005, Wang et al., 2006) which also uses the orthonormal propagation method. Seismic waveforms are automatically restituted to displacement, rotated into ZRT coordinates and filtered between 0.01 and 0.05Hz. Desired phases are automatically identified and tapered off. In this inversion we focus on surface waves only as waveforms at local distances were not available or clipped. The misfit calculation of the seismic waveforms based on the L^1 norm is performed in the time domain as it generally performs better for surface waves. Also it is rather sensitive to timeshifts (Kühn et al. 2020) that further helps to constrain the source location. We allow shifting between synthetic and observed data to capture inaccuracies in the velocity model (e.g., site-effects, topographic effects, lateral heterogeneities) but then applying a penalty function. In comparison to the other data domains that can be applied in Grond (e.g., frequency domain, cross-correlation, envelope), the absolute amplitude in the time domain directly contributes to the magnitude estimation.

The static displacements are forward modeled for each component (Z, N, E) of each GNSS station and compared with each of the components given in the observed data; the calculation of static deformation amplitudes is described in section 3.4. Here, we apply again the L^1 norm

for misfit calculation between observed (d_{obs}) and synthetic data (d_{synth}), that is then normalized by the data norm ($||e_0||$):

$$||e_{norm}|| = \frac{||e||}{||e_0||} = \frac{||\mathbf{d}_{obs} - \mathbf{d}_{synth}||}{||\mathbf{d}_{obs}||}$$

Depending on the source-receiver distance, phase types and station specific data processing (e.g., bandpass filter), individual stations might present stronger signal amplitudes and thus, higher contributions to the objective function. This is bypassed by the application of an adaptive station weighting (Heimann 2011) for balance weighting that enhances stations of small signals and suppresses stronger signals; the weighting factor is the inverse of mean signal amplitudes of synthetic waveforms randomly drawn from the model space. An additional weighting scheme is added based on the data error statistics using the inverse of the standard deviation. For the static displacements the 1-sigma uncertainty is directly derived from the time-correlated noise model fitted to the GNSS time series (for detail see section 3.4). The waveform errors are estimated from the data noise variance before the onset of the first phase arrival multiplied by the expected signal variance (Duputel, et al. 2012).

The global misfit for the final objective function is based on so-called normalization families, in order to separate the different natures of observations and their distinct relationships regarding the source parameters. Here, we use two normalization families that capture the static near-field observations from the GNSS data and the dynamic waveforms in the time domain. Although we invert for two different phases (Love and Rayleigh), these observations are not independent from each other. The global misfit ($e_{norm,global}$) is then defined as the root mean square of the individual normalized misfits of each normalization family:

$$||e_{norm,global}||_2 = \sqrt{\frac{\frac{||e_{time}||_2}{||e_{0,time}||_2} + \frac{||e_{static}||_2}{||e_{0,static}||_2}}{2}}$$

The Bayesian bootstrap optimization (BABO) is implemented as a paralleled Monte-Carlo random direct search. At first, a uniform random sampler explores the full extension of the predefined model space of possible source parameters. After 5,000 iterations it transitions into the directed sampling. This rather exploitative approach uses a continuously updated high score list of the best models based on the lowest misfit values. Within the 30,000 iterations, the search radius around the current high score models is repeatedly narrowed, starting with twice the standard deviation of the high score list of the uniform sampling, down to 0.5 the standard deviation. The parallelization is realized through 100 bootstrap chains, in which each chain consists of an independent objective function with distinct bootstrap weights but shared forward models. This step ensures a higher variety of high score models exploiting the model space in order to capture the model uncertainties given the data errors; the BABO can therefore properly sample the global minimum even in a multivariate posterior distribution.

References

Ammirati, J.-B., Alvarado, P., and Beck, S., 2015. A lithospheric velocity model for the flat slab region of Argentina from joint inversion of Rayleigh wave phase velocity dispersion and teleseismic receiver functions, Geophys. J. Int., 202, 224-241. https://doi.org/10.1093/gji/ggv140

Costa, C., 2021, Strong quake rattles San Juan, Temblor, http://doi.org/10.32858/temblor.154

Delouis, B., 2014. FMNEAR: Determination of focal mechanism and first estimate of rupture directivity using near‐source record and a linear distribution of point sources, Bull. Seismol. Soc. Am. 104(3), 1479–1500. doi: https://doi.org/10.1785/0120130151

Derode, B., Delouis, B., Campos, J., 2019; Systematic Determination of Focal Mechanisms over a Wide Magnitude Range: Insights from the Real‐Time FMNEAR Implementation

in Chile from 2015 to 2017. *Seismol. Res. Lett.* 90 (3), 1285–1295. doi: https://doi.org/10.1785

Duputel, Z., Rivera, L., Fukahata, Y. and Kanamori, H., 2012. Uncertainty estimations for seismic source inversions. Geophysical Journal International, 190(2), pp.1243-1256.

Geohazards TEP, Diapason S1 San Juan 120A, 2021. https://geohazards-tep.eu/t2api/share?url=https%3A%2F%2Fgeohazards-tep.eu%2Ft2api%2Fjob%2Fwps%2Fsearch%3Fid%3D17e25f2d-b4b3-4d61-a656-527068f6f598%26key%3Da6f3a55d-045f-4716-beba-305614c70af2 (last accessed July 2022)

Girino, G.S., Ortiz, G., Saez, M., López, L., Pérez, I., Moreno, M., Venerdini, A., Fernández, M. and Alvarado, P., 2021. El terremoto (Mw 6.4) superficial del 18 de enero de 2021 del Cordón de las Osamentas, Precordillera Central, San Juan, Argentina. *Revista de la Asociación Geológica Argentina*, *78*(4), pp.613-620.

Heimann, S., 2011. A robust method to estimate kinematic earthquake source parameters (Doctoral dissertation, Staats-und Universitätsbibliothek Hamburg Carl von Ossietzky).

Heimann, S., Vasyura-Bathke, H., Sudhaus, H., Isken, M. P., Kriegerowski, M., Steinberg, A., and Dahm, T., 2019. A Python framework for efficient use of pre-computed Green's functions in seismological and other physical forward and inverse source problems, Solid Earth, 10, 1921–1935, https://doi.org/10.5194/se-10-1921-2019.

Herrmann, R.B., 2013. Computer programs in seismology: An evolving tool for instruction and research. *Seismological Research Letters*, *84*(6), pp.1081-1088.

Kühn, D., Heimann, S., Isken, M. P., Ruigrok, E., and Dost, B., 2020. Probabilistic Moment Tensor Inversion for Hydrocarbon- Induced Seismicity in the Groningen Gas

Field, The Nether- lands, Part 1: Testing, Bull. Seismol. Soc. Am., 110, 2095–2111, https://doi.org/10.1785/0120200099

Laske, G., Masters, G., Ma, Z. and Pasyanos, M.E., 2012. CRUST1. 0: An updated global model of Earth's crust. *Geophys Res Abs*, *14*(3), p.743.

Venerdini, A., Sánchez, G., Alvarado, P., Bilbao, I. and Ammirati, J.B., 2016. Nuevas determinaciones de velocidades de ondas P y ondas S para la corteza sísmica del terreno Cuyania en el retroarco andino. *Revista mexicana de ciencias geológicas*, *33*(1), pp.59-71.

Wang, R., 1999. A simple orthonormalization method for the stable and efficient computation of Green's functions. - Bulletin of the Seismological Society of America, 89, 733-741.

Wang, R., 2005. The dislocation theory: a consistent way for including the gravity effect in (visco)elastic plane-earth models. - Geophysical Journal International, 161, 1, 191-196. https://doi.org/10.1111/j.1365-246X.2005.02614.x

Wang, R., Lorenzo Martín, F., Roth, F., 2006. PSGRN/PSCMP - a new code for calculating co- and post-seismic deformation, geoid and gravity changes based on the viscoelastic-gravitational dislocation theory. - Computers and Geosciences, 32, 4, 527-541. https://doi.org/10.1016/j.cageo.2005.08.006
